# Leveraging the molecular insights and therapeutic potential of diet-derived-flavonoids against pancreatic ductal adenocarcinoma

**DOI:** 10.3389/fphar.2026.1864116

**Published:** 2026-08-10

**Authors:** Sivaramasundaram Sankarasubramanian, Naveenkumar Perumal

**Affiliations:** School of Bio Sciences and Technology, Vellore Institute of Technology, Vellore, Tamil Nadu, India

**Keywords:** apoptosis, cell cycle arrest, chemoresistance, dietary flavonoids, oxidative stress, PDAC

## Abstract

**Introduction:**

Pancreatic ductal adenocarcinoma (PDAC) remains one of the most lethal malignancies of the gastrointestinal tract, characterized by its subtle onset, aggressive progression, and poor prognosis. Due to intrinsic resistance to drugs and high relapse rates after conventional therapies, the need to investigate alternative therapeutic approaches with improved efficacy and tolerability.

**Methods:**

An extensive literature review was carried out through major scientific databases to spot relevant studies on different classifications of flavonoids and their anti-cancer potential in PDAC.

**Results:**

Recent research demonstrates the potential of bioactive substances derived from plants, notably flavonoids, as modulators of oncogenic signaling networks with potent anti-cancer properties, such as inhibition of oxidative stress, angiogenesis, and other essential regulatory mechanisms associated with the development of tumors. Research has shown the molecular mechanisms of flavonoids across a wide range of cancer hallmarks, including inflammation, immunological modulation, redox homeostasis, cell proliferation, apoptosis, autophagy, and cell cycle control.

**Discussion:**

This review provides current insights into the molecular targets and signaling pathways that flavonoids influence against PDAC, making them appealing for either standalone or supplementary treatment.

## Graphical Abstract

**Figure:**
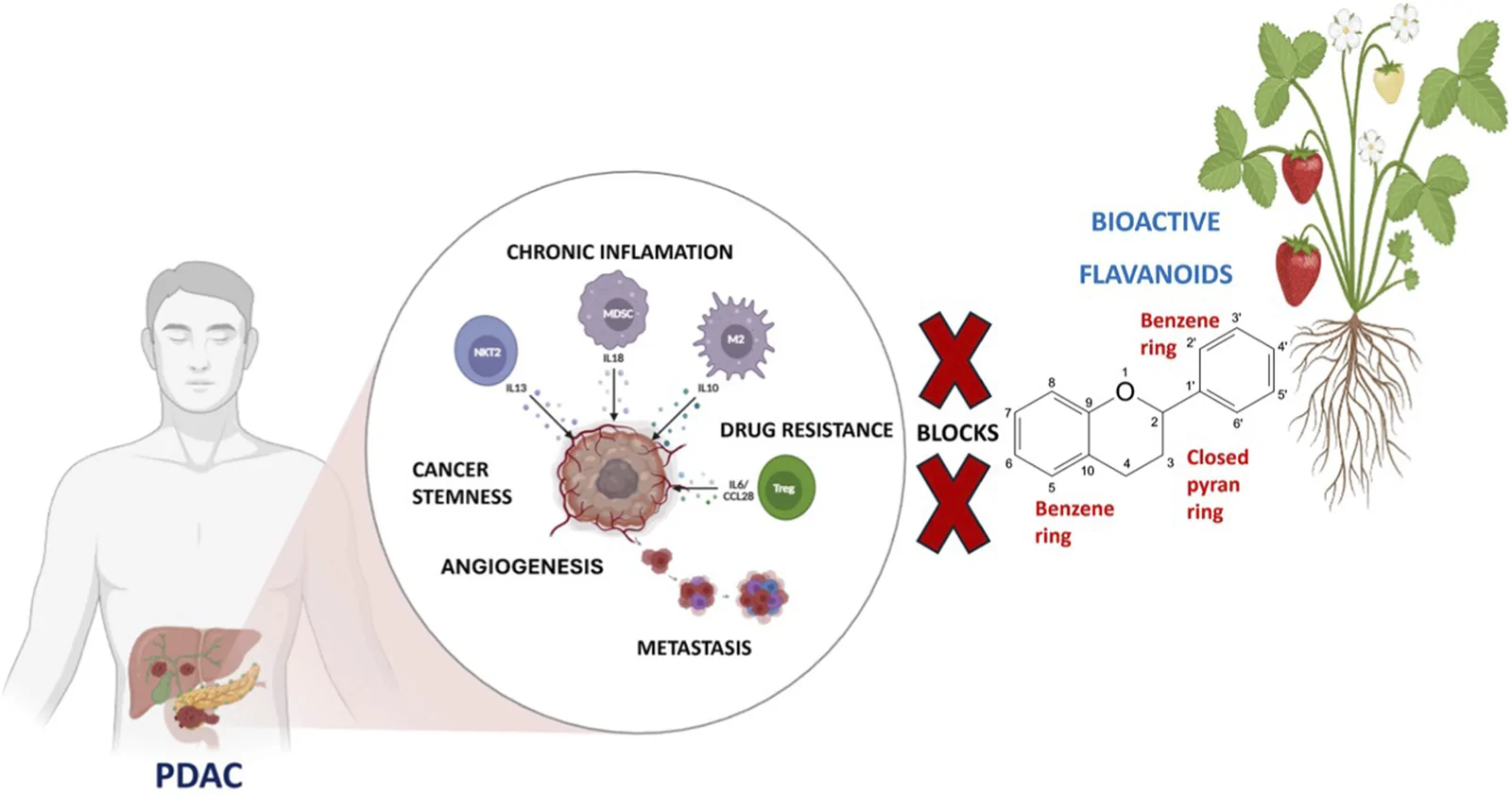

## Highlights

- PDAC is a gastrointestinal cancer with poor clinical results that is extremely aggressive and frequently detected late.
- Dietary flavonoid-based plant therapeutics show significant anti-cancer activity in preclinical and clinical trials and have minimal toxicity.
- Flavonoids exhibit a wide range of bioactivities, including the ability to modulate immunological responses, reduce inflammation, and act as anti-oxidants.
- A promising new treatment option for PDAC worldwide is the combination of flavonoids with traditional chemotherapy medications.

## 1 Introduction

Gastrointestinal cancers include esophagus, stomach, liver, bile duct, gall bladder, pancreas, and intestinal malignancies, representing 26% of total cancer diagnoses. Specifically, pancreatic tumors have an incidence rate of 2.5% and contribute to 4.5% mortality globally (Arnold et al., 2020). Pancreatic cancers rank 11th globally and 24th in India, with an 18% mortality rate (Rawla et al., 2019). Likewise, the high incidence rate of pancreatic cancer varies across North America, Europe and high human development index countries, while South Asia and Africa have lower rates. About 1% of all cancer cases in India are pancreatic cancer, with a higher incidence reported in northeastern regions (Gaidhani and Balasubramaniam, 2021). Certain ethnic groups, such as Africans and Americans, have greater incidence rates, with men slightly more susceptible than women (Hu et al., 2021; Mack et al., 2025). Smoking, obesity, diabetes, chronic pancreatitis, alcohol use, eating habits, advanced age, and genetic susceptibility are major risk factors of pancreatic cancer (Mizrahi et al., 2020). Pancreatic cancer is also associated with poor prognosis and 5 years overall survival rate of less than 10%. The lack of efficient targeted treatments and an innate resistance to chemotherapeutic medications are the primary causes for poor prognosis and high mortality in pancreatic cancer (Suhail et al., 2025). The increasing prevalence of pancreatic cancer worldwide underscores the critical need for better early detection methods and the development of more potent therapeutics.

Pancreatic ductal adenocarcinoma (PDAC) is the most predominant form of pancreatic cancer and originates from the exocrine part of the pancreas. PDAC progresses slowly and remains asymptomatic until advanced stages, and is often sporadic and typically occurs later in life. Due to poor prognosis, survival rates remain low, i.e., only 24% at 1 year and 9% at 5 years (Rawla et al., 2019). Diagnostic evaluation depends upon CT imaging with high sensitivity (90% sensitivity) and serum biomarkers, including CA19-9, CEA, and CA125 (Yamao et al., 1999). The pathophysiology of PDAC is mainly driven by genetic mutations and epigenetic alterations, leading to the transformation of pancreatic ductal epithelial cells into carcinoma (Montalvo-Javé et al., 2023). Over 90% of PDAC patients have mutations in KRAS gene that result in tumor formation (Mueller et al., 2018). Mutations occurring in TP53, CDKN2A, and SMAD4 contribute to abnormal cellular proliferation, survival of the cells despite apoptotic signals, chromosomal instability, and metastasis (Helen et al., 2024; Stefanoudakis et al., 2024). Gut microbiota dysregulation also contributes to PDAC, with elevated *Porphyromonas gingivalis* and *Granulicatella adiacens*, and reduced *Neisseria elongata* and *Streptococcus mitis*, linked to higher cancer risk (McGuigan et al., 2018). Consequently, these cancer-causing changes lead to the triggering of several malignant pathways like MAPK, PI3K/Akt, TGF-β, Wnt/β-catenin, and NF-κB signaling that cooperate in tumor progression and survival (Murthy et al., 2018; Du et al., 2026). Also, PDAC undergoes massive metabolic reprogramming through increased glycolysis and glutamine metabolism as well as autophagy to adapt and maintain growth in the nutrient-depleted microenvironment (Li et al., 2021). PDAC is characterized by its asymptomatic nature, resistance to conventional therapies, high relapse rates, and cancer stem cell-like properties, making it as one of the aggressive cancer types complicating effective treatment (Nimmakayala et al., 2022; Bhatia et al., 2023). Despite conventional treatment options in PDAC like surgical resection, chemotherapy, and radiotherapy, therapeutic efficacy remains limited due to high relapse rates and low survival outcomes. The first-line treatment option for PDAC is Gemcitabine, followed by paclitaxel and other drugs, but these drugs have failed to improve survival and present adverse side effects (Adamska et al., 2017). To overcome and address these limitations, plant-based bioactive compounds may provide potential alternatives, either as an adjunct to traditional treatment or as a stand-alone treatment. The stromal microenvironment of PDAC is notable for its cellular and spatial variability, which significantly impacts treatment resistance and disease biology. Efforts to better understand the various cell types and acellular characteristics in the PDAC microenvironment and how they affect tumor progression have been hampered by its complexity. As cancer cells interact with host structural elements, the tumor microenvironment (TME) undergoes a dynamic transformation that includes cellular and molecular changes (Sherman and Beatty, 2023). Several hallmarks of cancer, such as angiogenesis, apoptosis inhibition, immunomodulation, invasion, and hypoxia are directly associated with TME. Therefore, modifying the TME may be an effective strategy for treating and preventing cancer. Flavonoids are known to inhibit angiogenesis, reduce inflammation, induce apoptosis and modulate the tumor immune microenvironment (TIME). Due to its crucial role in the genesis of cancer, targeting TME with flavonoids provides a promising hope in cancer therapy (Saadh et al., 2024).

Also recently, Flavonoids are the reformers in PDAC treatment due to their low cost, less toxicity, high bioavailability and improved therapeutic efficacy (Casarcia et al., 2025; Lee et al., 2025). Although flavonoids such as apigenin, genistein, quercetin, and naringenin show promising anti-cancer activities, they exhibit low bioavailability that hinders their clinical translation. Low aqueous solubility, minimal intestinal absorption and rapid metabolism have been attributed to their limited bioavailability. However, various strategies, including structural modification, nano formulations and liposomal-mediated delivery systems, are being explored currently to enhance bioavailability and therapeutic efficacy.

## 2 Types and treatment of pancreatic cancer

**FIGURE 1:**
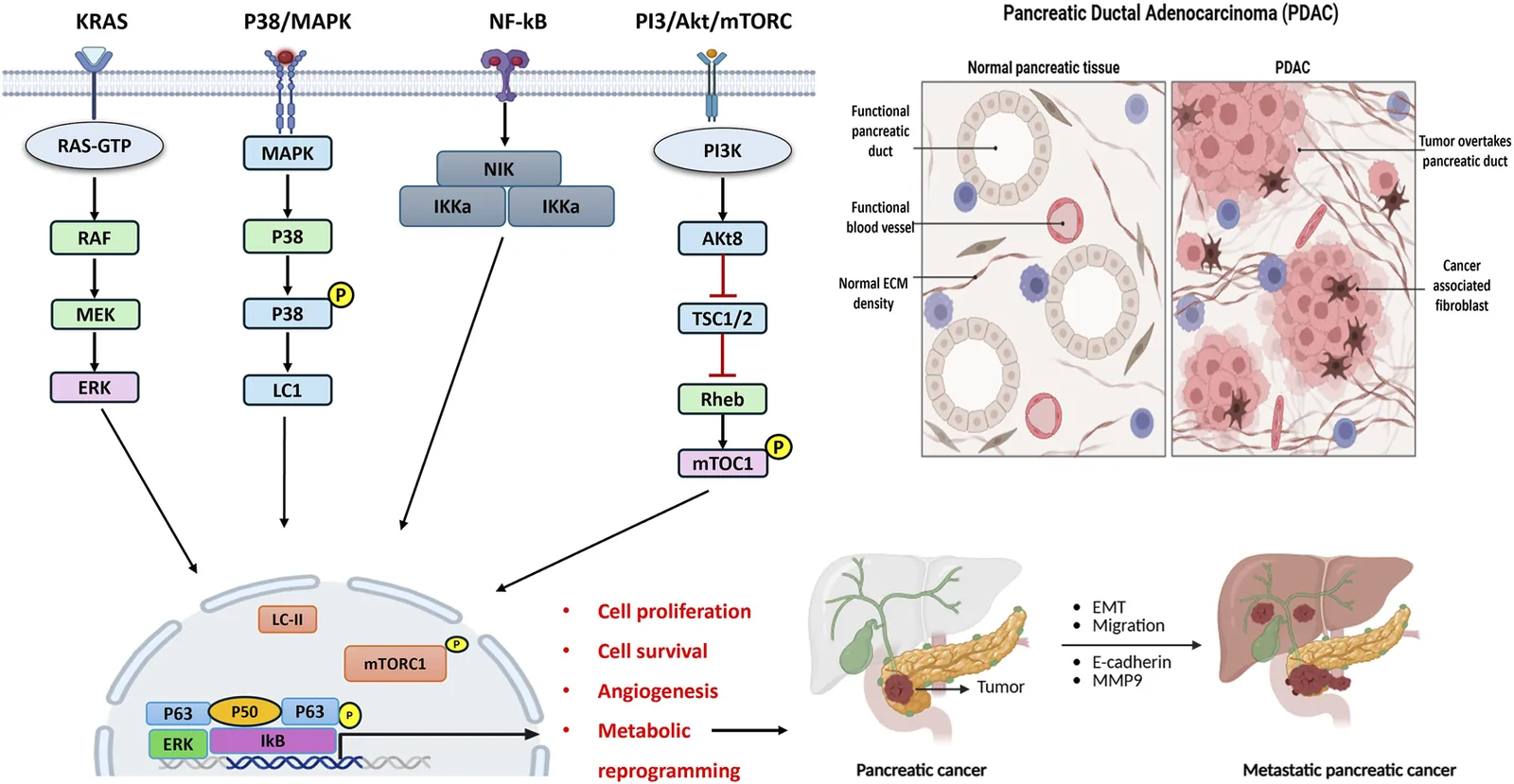
Overall Representation of Pancreatic Ductal Adenocarcinoma. The figure depicts the tumor progression, highlighting the key oncogenic drivers at various stages of Pancreatic ductal adenocarcinoma (PDAC).

Pancreatic cancer is categorised into two primary types as exocrine and endocrine, where the exocrine tumor makes up to 95% of all cases and is known for its aggressive behaviour (Zhou et al., 2010). Among exocrine malignancies, PDAC is the most aggressive form, characterized by its asymptomatic progression and lack of reliable early-stage biomarkers, thereby imposing significant challenges to timely diagnosis, which impacts the devising of effective therapeutic strategies (Rawla et al., 2019). In addition to becoming resistant to chemotherapy, exhibiting cancer stemness and high relapse rates, PDAC usually displays at the last stage, when surgical resection is nearly impossible. Gene mutations such as KRAS, CDKN2A, SMAD4, and TP53 modulate the tumor microenvironment of PDAC, contributing to the poor therapeutic outcomes (Saiki et al., 2021; Chugh et al., 2018). Figure 1 depicts the oncogenic drivers, symptoms, diagnosis, stages, and available treatments for PDAC. Endocrine pancreatic cancer is also quite common, with an incidence rate of 1% (Haugvik et al., 2016). Diabetes is a significant risk factor for either spontaneous or hereditary pancreatic neuroendocrine tumors (PNET) (Panzuto et al., 2011). A multifactorial-based therapeutic approach to pancreatic cancer is needed that is reliant upon the disease stage, histological subtypes, patient age and general physiological health.

Management strategies encompass a spectrum of modalities, including systemic chemotherapy, radiation, surgical resection, and immunotherapeutic treatments (Mehtsun et al., 2019; Falconi, 2021; Principe et al., 2021). The first drug approved by the FDA for the treatment of PDAC is gemcitabine. When combined with paclitaxel and FOLFIRINOX were shown to increase disease-free survival (Principe et al., 2021). Also, FDA-approved treatments such as immune checkpoint antibodies and engineered T-cell therapy for other cancers show minimal efficacy in pancreatic cancer (Paredes-Moscosso and Nathwani, 2024). Ongoing trials on checkpoint inhibitors, adoptive cell transfer and cancer vaccines are being investigated to conjugate with the existing molecular therapies and chemotherapy (Johansson et al., 2016). Monoclonal antibodies such as Anetumab and maytansine, which could serve as a second-line treatment, are also under investigation (Passero and Saif, 2017).

## 3 Dietary flavonoids in PDAC treatment

Lifestyle changes and dietary food habit changes have led to an increase in chronic diseases such as diabetes, obesity and cancers (Zhang et al., 2025). Oncology research has shifted its focus towards natural compounds made from medicinal plants due to their wide range of therapeutic possibilities, especially the significant anti-cancer activity. These natural bioactive compounds offer lower toxicity, more bioavailability, and enhanced therapeutic efficacy compared to the conventional therapies (Pavithra et al., 2024). Numerous categories of phytochemicals, like flavonoids, polyphenols, terpenoids, and alkaloids, have had their anti-cancer properties extensively studied (Dariya et al., 2019). Polyphenolic compounds, particularly flavonoids, have exhibited significant pharmacological potential in the context of pancreatic cancer, primarily through modulation of key molecular signaling cascades (Wan et al., 2024).

Flavonoids constitute a diverse group of polyphenolic secondary metabolites, ubiquitously distributed across a wide array of dietary sources, including fruits, vegetables, and plant-derived beverages. The highest level of flavonoids is present in berries, plums, sweet cherries and apples. Flavonoids and their polymers form a large group of food constituents that help to positively impact health, thereby altering metabolic pathways (Liang et al., 2025). Flavonoids in food occur mostly as tannins, condensed tannins, derived tannins, and hydroxy stable tannins as large polymers. The frequent intake of fruits and vegetables that are enriched with flavonoids has been recommended to prevent diseases, including cancer. Recent studies on flavonoids’ role in cancer have shown that intake of flavonoids is inversely associated with cancer mortality (Kubatka et al., 2021). Unbalanced anti-oxidant mechanisms and pro-oxidant load lead to the accumulation of reactive oxygen species and generation of free radicals. This oxidative stress is closely related to the development of many diseases and cancer. Therefore, nutritious food, loaded with anti-oxidants, is necessary to maintain cellular homeostasis. Flavonoids can scavenge reactive oxygen species due to the presence of a phenolic hydroxyl group, thereby exhibiting their potent anti-oxidant activity (Treml and Šmejkal, 2016). Flavonoids attenuating the hallmarks of PDAC are shown in Table 1.

**TABLE 1:** Representation of flavonoids in inhibiting the hallmarks of PDAC.

S.No	Hallmarks of PDAC	Flavonoids	References
1	Cell proliferation	Hesperidin, Cyanidin	Lee et al. (2019), Farhan et al. (2022)
2	Cancer stemness	Quercetin, Genistein	Cao et al. (2015), Bao et al. (2011)
3	Chemoresistance	Apigenin, Anthocyanin	Stern et al. (2022), Mostafa et al. (2023)
4	Angiogenesis	Luteolin, Catechins	Pan et al. (2026), Shankar (2008)
5	Invasion and migration	Naringenin, Genistein	Lou et al. (2012), Xia et al. (2014)
6	Anti-apoptosis	Myricetin, Kaempferol	Phillips et al. (2011), Wang et al. (2021)

**FIGURE 2:**
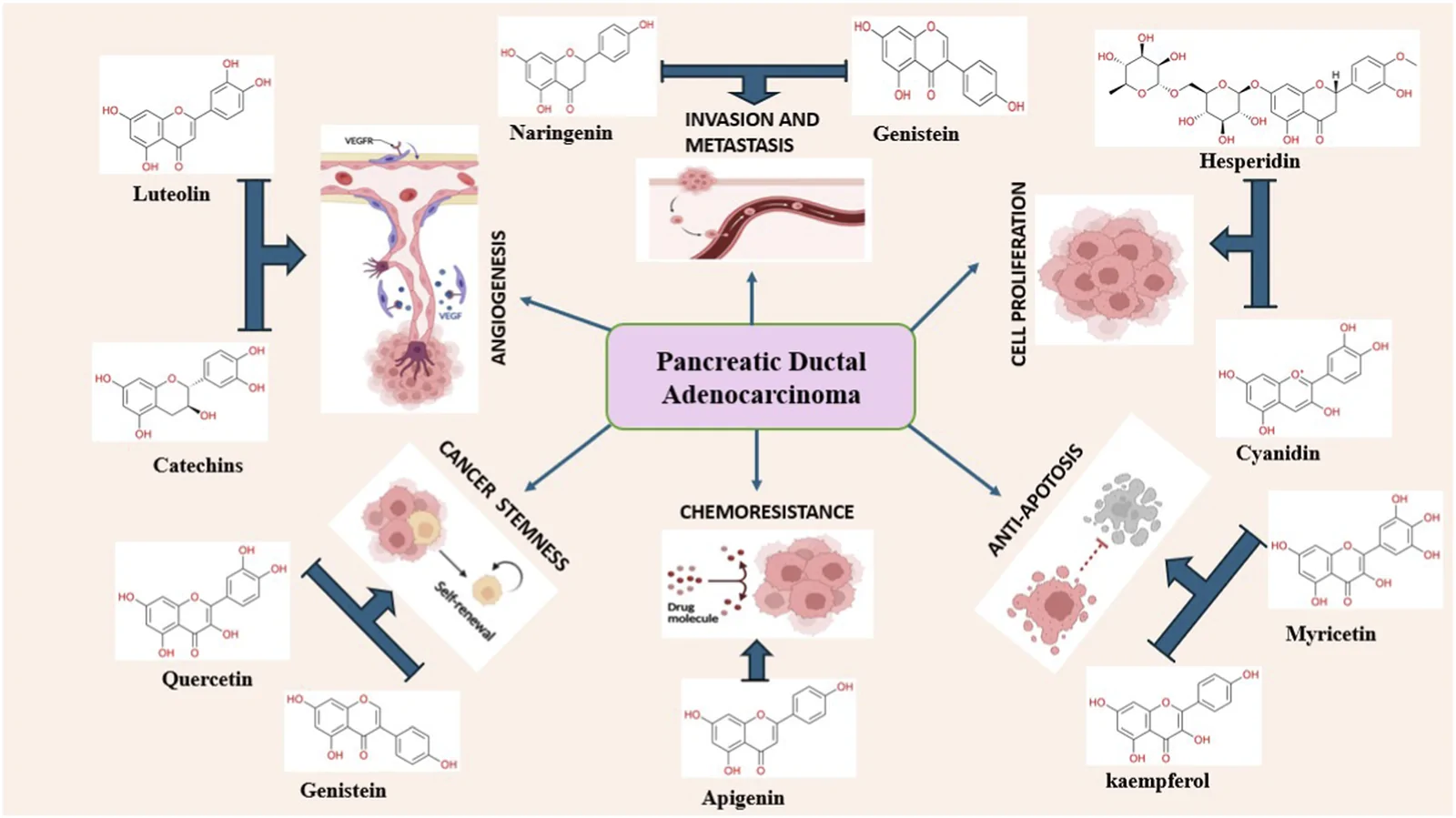
Flavonoids targeting hallmarks of PDAC. The figure depicts the flavonoids, a diverse group of phytochemicals found in plants, including apigenin, hesperidin, cyanidin, myricetin, kaempferol, anthocyanins, quercetin, catechins, luteolin, naringenin and genistein targeting the hallmarks of PDAC such as cell proliferation, angiogenesis, invasion and metastasis, anti-apoptosis, cancer stemness, and chemoresistance.

**FIGURE 3:**
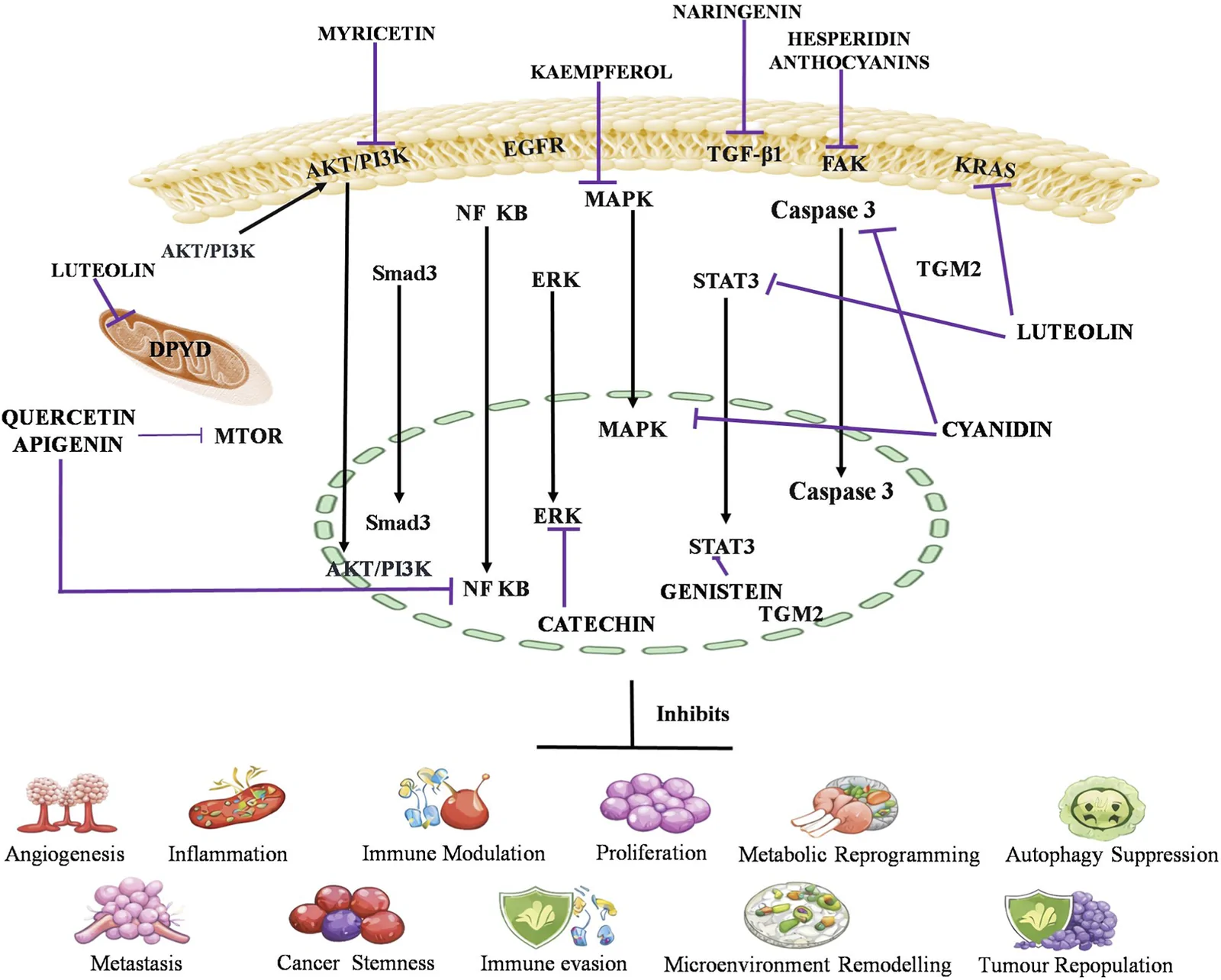
Dietary flavonoid-mediated regulation of the molecular oncogenic signaling pathways against PDAC. The figure shows the molecular mechanism through which flavonoids regulate the intracellular pathways during cancer progression. Flavonoids, including apigenin, quercetin, genistein, catechins, hesperidin, myricetin, anthocyanins, naringenin and kaempferol, show interaction with the receptors and signaling cascades such as AKT/P13K, TGF-β1, KRAS, EGFR, mTOR signaling, leading to the inhibition of cell proliferation, angiogenesis, inflammation, immune modulation, metabolic reprogramming, autophagy suppression, cancer stemness, immune invasion, tumor repopulation, tumor microenvironment remodelling and metastasis.

Flavonoids are notable for their capacity to inhibit tumor growth, regulate oxidative stress, and interfere with cancer-promoting signaling cascades (Figure 2). The modulation of molecular targets via flavonoids is depicted in Figure 3. Their broad bioactivity spectrum includes anti-inflammatory, anti-angiogenic, apoptotic, and immunomodulatory properties, making them highly relevant in PDAC management (Farhan et al., 2023). Flavonoid-based adjunct or alternative therapies have been made possible by experimental investigations that have shown flavonoids' ability to improve chemotherapeutic efficacy, decrease drug resistance, and attenuate tumor metastasis. Some flavonoids, including apigenin and myricetin, exhibited strong anti-oxidant properties, contributing to their chemopreventive and therapeutic potential across a spectrum of malignancies, such as breast, pulmonary, colorectal, pancreatic, and gastric cancers (Farhan et al., 2023). Flavonoids affecting molecular oncogenic signaling pathways involved in PDAC cancer progression and their combined effect with the other drugs are listed in Tables 2, 3.

**TABLE 2:** Representation of class, source, molecular target, signaling pathway, mechanism of action and therapeutic effects of flavonoids in PDAC.

S.No	Class of flavonoids	Flavonoids	Source	Molecular target	Signaling pathways	Mechanism of action	Therapeutic effects	References
1	Flavonols	Quercetin	Fruits and vegetables (onion, apples, red leaf lettuce, broccoli, and cherries)	NF-κB, PI3K/Akt/mTOR	NF-κB, GSK-3β and mTOR/Akt/PI3K	Inhibits NF-κB signaling; Induces apoptosis and autophagy; Reduces cell survival and proliferation	Anti-proliferative, pro-apoptotic and inhibition of tumor growth	Cao et al. (2015), Nwaeburu et al. (2016), Yu et al. (2017)
		Myricetin	Azadirachta indica, nuts, and wine	PI3K, Akt	PI3K/Akt	Inhibits PI3K signaling, reduces cell survival and proliferation	Anti-proliferative, pro-apoptotic and suppression of tumor growth	Javed et al. (2022)
		Kaempferol	Vegetables (kale, spinach, and broccoli), and fruits (apples, grapes, and strawberries)	TGM2, EGFR	TGM2-mediated Akt/mTOR, PI3K/Akt, EGFR and MAPK.	Modulates ROS-mediated apoptosis through TGM2-dependent signaling; Inhibits EGFR signaling, reduces cell growth, migration, and metastasis	Anti-proliferative, pro-apoptotic and anti-migratory effects	Lee and Kim (2016), Wang et al. (2021)
		Morin	Moraceae plants, almonds, bilberries, and Cudrania tricuspidata bread leaves	PINK1/Parkin	PINK1/Parkin-mediated mitophagy pathway	Activates PINK1/Parkin-mediated mitophagy, leading to elimination of damaged mitochondria and inhibition of tumor growth	Induction of mitophagy and tumor suppression	Sharma et al. (2026)
2	Flavones	Apigenin	Parsley and the dried inflorescences of chamomile	GSK-3, IKK-β	PI3K/Akt, GSK-3/NF-κB, IKK-β-mediated NF-κB inhibition	Blocks GSK-3/NF-κB signaling, promoting mitochondrial-mediated apoptosis; Suppresses IKK-β activity, preventing activation of the NF-κB pathway	Induction of apoptosis, and inhibition of cell proliferation and tumor growth	Johnson and de Mejia (2013), Feng et al. (2023)
		Luteolin	Peppers, thyme, celery, parsley, broccoli, etc.	KRAS, MEP1A, DPYD, STAT3	KRAS/GSK-3β/NF-κB, DPYD, MEP1A and STAT3	Inhibits KRAS mediated signaling; Decreases GSK-3β signaling; Suppresses MEP1A expression associated with PDAC aggressiveness	Reversal of chemoresistance, pro-apoptotic, reduces invasion and metastasis	Kato et al. (2024), Rauf et al. (2024), Pandey et al. (2025)
3	Flavanones	Hesperidin	Citrus fruits (Oranges, and lemons)	MMK3/6, FAK, P38 MAPK	MKK3/6, FAK and P38	Modulates the MKK3/6–p38 signaling pathway, attenuates migration and survival signaling; Inhibits FAK signaling, reducing cell adhesion, migration, and invasion; Downregulates p38 signaling suppressing tumor growth and metastatic potential	Inhibition of cell proliferation, invasion, migration and reduced tumor progression	Aja et al. (2020), Lee et al. (2019)
​	​	Naringenin	Citrus fruits (Grapes)	Prdx-1, TGF-β1, Smad3	Prdx-1, TGF-β1/Smad3	Inhibits Prdx-1, a protein involved in cell survival and proliferation, resulting in apoptotic cell death; Suppresses TGF-β1 signaling, reducing invasion, metastasis, and gemcitabine resistance; Inhibits TGF-β1/Smad3 signaling, leading to decreased EMT.	Induction of apoptosis; Inhibition of EMT and chemoresistance; Anti-metastatic effects	Park et al. (2017), Lin et al. (2023)
		Eriodictyol	Citrus species and Cereal grains	Akt/ERK	Akt/ERK	Suppression of Akt and ERK phosphorylation, coupled with enhanced activation of the JNK signaling pathway	Induction of apoptosis and cytotoxic cell death	Islam et al. (2020), Oh et al. (2022)
4	Flavanols	Catechins	Tea leaves and several fruits	NF-κB (p65 subunit), PI3K/Akt, FKHRL1/FOXO3a	NF-κB, ERK, PI3K/AKT, FKHRL1/FOXO3a and JNK	EGCG binds to the p65 subunit of NF-κB, inhibiting its activity and promoting apoptosis; Suppresses PI3K/Akt signaling, reducing cell survival signaling; Activates FOXO3a transcription factor, promoting apoptosis and cell cycle regulation	Anti-proliferative and pro-apoptotic effects	Qanungo et al. (2005), Kürbitz et al. (2011), Kostin et al. (2012), Michel et al. (2018)
5	Isoflavones	Genistein	Soybeans	STAT3, P13K, NF-κB	STAT3, Smad4, P38 MAPK/P13K, Akt and NF-κB	Inhibits STAT3 phosphorylation in a dose-dependent manner, suppressing tumor growth and cell survival signaling; Suppresses NF-κB activation, reducing proliferation, survival, and metastatic signaling	Anti-proliferative and pro-apoptotic effects; Reduced cell survival and proliferation; Anti-inflammatory and anti-tumor effects	El-Rayes et al. (2006), Löhr et al. (2016), Ma et al. (2016)
		Anthocyanins	Fruits and Vegetables	NF-κB (p65), FAK	NF-κB p65 and FAK	Suppresses NF-κB signaling, reducing migration, invasion, and tumor progression; Reduces FAK phosphorylation, impairing cancer cell motility and metastasis	Anti-inflammatory and anti-metastatic effects; Inhibition of migration and cell adhesion	Kuntz et al. (2017), Mostafa et al. (2023)

**TABLE 3:** Representation of the synergistic effects of flavonoids with chemotherapeutic drugs.

S.No	Combined therapy	Synergistic effects	References
1	Quercetin and Gemcitabine	The combination of these two reduces the expression of beta-catenin and improves the prognosis of PDAC. Also, quercetin leads to S-phase arrest in GEM-resistant cells, leading to apoptosis	Cao et al. (2015), Liu et al. (2020)
2	Kaempferol + Gemcitabine or 5-FU	Kaempferol shows an additive effect and can be used as a combination drug with gemcitabine or 5-FU for better therapeutic efficacy	Zhang et al. (2008)
3	A) Apigenin + Gemcitabine B) Apigenin + 5-FU	A) The combination of gemcitabine with apigenin enhanced tumor suppression by mediating Akt and NF-κB activity B) Apigenin enhanced the anti-proliferative effect of 5-FU by GSK-3β/NF-κB signaling pathway	Lee et al. (2008)
4	Luteolin + 5-FU and Gemcitabine	The combined treatment of luteolin with 5-FU and gemcitabine inhibits PDAC by inhibiting the expression of DPYD.	Johnson et al. (2015), Kato et al. (2024)
5	Hesperidin + Naringenin	The combination of these two flavonoids induced apoptosis by inducing caspase-3 cleavage	Lee et al. (2019)
6	Catechins + Quercetin	Different forms of catechin (EGCG, EG, CG) effectively attenuated the self -renewal property as well as the migratory potential of the pancreatic cancer cells	Appari et al. (2014)
7	Genistein + Erlotinib and Gemcitabine	Investigations into combinatorial therapeutic strategies have revealed that genistein potentiates the efficacy of erlotinib and gemcitabine in pancreatic cancer cell lines by suppressing EGFR-independent activation of the Akt/NF-κB signaling axis. This synergistic interaction effectively lowers the apoptotic threshold, thereby enhancing cell susceptibility to programmed death and offering a promising approach to overcoming chemoresistance	El-Rayes et al. (2006)

### 3.1 Flavonols

#### 3.1.1 Quercetin

Quercetin is a widely distributed, naturally occurring flavanol, predominantly present in a variety of fruits and vegetables (onion, apples, red leaf lettuce, Broccoli, and cherries) with the ability to exert anti-fungal, anti-oxidant, cytotoxic, hepatoprotective, anti-inflammatory, and anti-cancer activities through inhibition of key oncogenic pathways (Baqer et al., 2024). Quercetin and other bioactive compounds present in the extracts of S. *melongena* and *D. orientalis* show apoptotic, anti-invasive and anti-migratory effects on pancreatic cancer cell lines (Yildirim et al., 2025). Quercetin exerts its anti-neoplastic effects through the modulation of key oncogenic signaling pathways, including NF-κB, GSK-3β, and the mTOR/Akt/PI3K axis. Their interactions facilitate a range of tumor-suppressive mechanisms, such as the induction of autophagy and apoptosis, enforcement of cell cycle arrest, and inhibition of metastatic progression (Angst et al., 2013; Davoodvandi et al., 2020). The unsurpassed anti-oxidant ability of quercetin helps to prevent oxidative stress via neutralizing the free radicals and cancer development. The natural form of quercetin glucosides is absorbed easily by the intestine through oral administration, which leads to the subsequent diffusion and transport via sodium-dependent glucose transporter, resulting in the subsequent attenuation of pancreatic cancer growth *in vivo* (Angst et al., 2013). Also, studies on the orthotopic animal model showed reduced tumor growth when the animals are treated with the different isoforms of quercetin, such as quercetin aglycones, compared with quercetin glucosides, suggesting the significance of quercetin isoforms in the treatment of pancreatic cancer (Angst et al., 2013). Differential gene expression studies support that β-Catenin plays a supportive role in pancreatic cancer maintenance and progression. Consequently, silencing β-Catenin expression via quercetin treatment leads to a reduction in its levels within pancreatic tumor stem-like cells relative to normal counterparts, thereby suppressing cellular proliferation and enhancing apoptotic activity in both *in vivo* and *in vitro* models (Cao et al., 2015).

**FIGURE 4:**
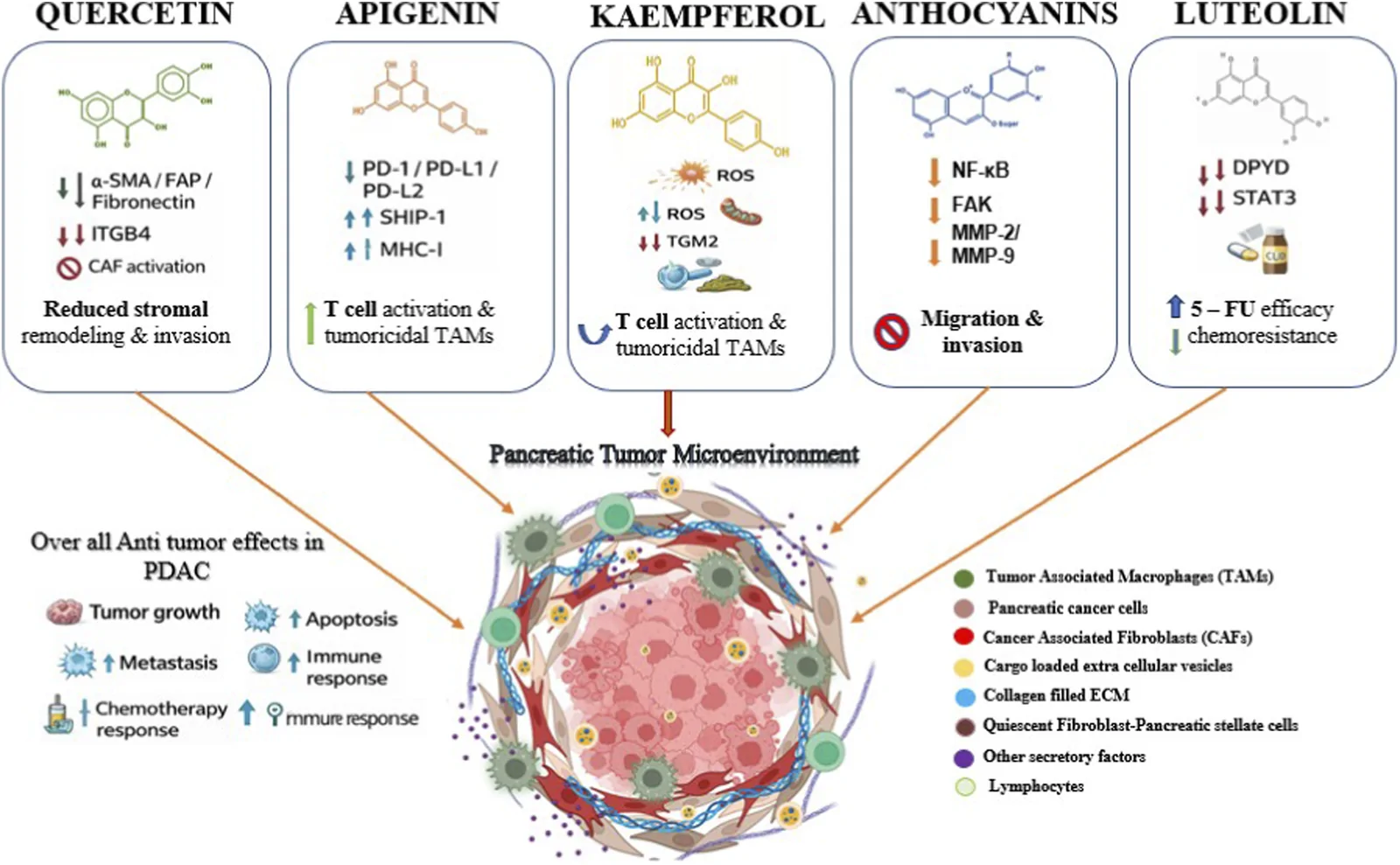
Flavonoids modulating Tumor Microenvironment in PDAC. The figure depicts the involvement of flavonoids such as quercetin, apigenin, kaempferol, anthocyanins and luteolins in modulating tumor microenvironment-associated signaling pathways. These compounds contribute to reduced stromal infiltration, migration, invasion, and chemoresistance while enhancing T-cell activation and tumor-associated macrophages (TAMs), thereby attenuating PDAC tumour growth, metastasis and recurrence.

The tumor microenvironment plays a vital role in PDAC progression, metastasis and chemoresistance. Cancer-associated fibroblasts (CAFs) are referred to as the most dominant and active stromal cells in TME of PDAC, which leads to the contribution of matrix deposition, tumor metastasis, and resistance to drugs upon activation. This activation can also lead to the stromal deposition and collagen remodelling that further accelerates the progression of tumor cells (Sahai et al., 2020; Rebelo et al., 2023). Flavonoids modulating tumor microenvironment in PDAC are depicted in Figure 4. Quercetin regulates the TME in PDAC and attenuates the activation of cancer-associated fibroblasts through tumor-stromal interaction. Also, bioinformatic analysis, such as RNA sequencing analysis, shows that quercetin is also involved in the infiltration of stromal cells in PDAC (Lei et al., 2024). Researchers have found that quercetin was able to suppress CAF activation in PDAC cells by downregulating the *α*-SMA, FAP, and Fibronectin expression. ITGB4 is highly expressed in various cancers, which plays a major role in CAFs and tumor cell interaction. Also, the high expression of this gene is associated with increased tumor invasion, drug resistance and poor prognosis among PDAC patients. Treating PDAC cells with quercetin in a dose-dependent manner reduces ITGB4 at protein and mRNA levels, implicating that it is a downstream target of quercetin (Lei et al., 2024).

Microarray analysis identified differential expression of 105 microRNAs (miRNAs) between quercetin-treated and untreated pancreatic cancer cells. Among these, the expression of the tumor suppressor miRNA let-7c, which is generally downregulated in pancreatic cancer, increased after quercetin treatment. Prior research has shown that quercetin directly interacts with the 3′untranslated region (3′-UTR) of NUMBL to activate transcription and cause let-7c expression. In addition to increasing apoptosis, this interaction inhibits the growth, progression, and metastasis of tumors by antagonistically interacting with the Notch signaling system (Nwaeburu et al., 2016). Additionally, it has been shown that combinatorial treatment regimens that include sulforaphane or quercetin in addition to green tea catechins (GTCs) have been demonstrated to attenuate KRAS oncogene expression through the upregulation of miR-let-7a, thereby contributing to molecular suppression of tumorigenic signaling (Appari et al., 2014).

The receptor for advanced glycation end products (RAGE) has been implicated as a key mediator in the pathobiology of pancreatic cancer, linked to tumor growth, metastasis, and treatment resistance, particularly gemcitabine resistance in afflicted patients (Lan et al., 2019). Quercetin treatment in MIA PaCa-2 targets RAGE, reducing its expression, thereby promoting cell cycle arrest, apoptosis, autophagy, and GEM chemosensitivity. Silencing of RAGE gene expression was reported to upregulate LC3-II protein levels and downregulate vital components of the PI3K/Akt/mTOR signaling cascade, which in turn promoted autophagic activity in MIA PaCa-2 pancreatic cancer cells (Lan et al., 2019).

Scientific evidence suggests that administration of quercetin reduced c-Myc expression significantly and prevented the epithelial-Mesenchymal transition (EMT) process, mainly by downregulating Transforming Growth Factor beta 1 (TGF-β1) signaling. These molecular modulations suppress oncogenic cellular behaviour, such as uncontrolled cell proliferation, migratory ability, and invasive potential (Guo et al., 2021). Pancreatic cells secrete elevated levels of interleukin-6 (IL-6), which triggers STAT-3 signaling pathway, promoting pancreatic intraepithelial neoplasia, aiding in the development of cancer. But when cells are treated with quercetin, they effectively counteract IL-6-mediated EMT by inhibiting STAT3 signaling pathways. This holistic approach promotes quercetin as a promising therapeutic agent in the treatment of pancreatic cancer (Yu et al., 2017). It has been shown that quercetin and its isoforms, quercetin 3-O-glucoside and quercetin 7-O-glucoside, block epidermal growth factor receptor (EGFR) signaling in a dose-dependent manner. Among these, quercetin 3-O-glucoside exhibits pronounced suppression of EGFR-mediated downstream pathways, including focal adhesion kinase (FAK), Akt, MEK1/2, and ERK1/2. Notably, its co-administration with gemcitabine elicits a synergistic anti-migratory response in PDAC cell lines (Lee J. et al., 2015). Quercetin is also a prospective therapeutic option for individuals who are resistant to gemcitabine-based regimens because of recent findings showing that it can induce S-phase cell cycle arrest, stimulate apoptotic processes, and increase p53 expression (Liu et al., 2020).

#### 3.1.2 Myricetin

Myricetin a bioactive phytochemical flavonoid 3,5,7,3′,4′,5′-hexahydroxyflavone is mostly found in a wide range of fruits, vegetables, and leafy plant materials. It is particularly found in *Azadirachta indica*, which shown to have a wide range of biologically active properties, including anti-inflammatory, anti-diabetic, anti-oxidant and anti-cancer effects (Ozcan and Yaman, 2015; Almatroodi and Rahmani, 2025; Trivedi et al., 2024). According to Wang et al. (2010), Myricetin’s strong anti-oxidant property scavenges the intracellular reactive oxygen species (ROS) and restores the protein expression and enzymatic activity of essential anti-oxidant Défense systems, such as glutathione peroxidase (GPx), catalase (CAT), and superoxide dismutase (SOD). In addition, Myricetin shows pro-apoptotic activity in pancreatic cancer cells, which is due to the inhibition of the phosphoinositide 3-kinase (PI3K) signaling axis and concurrent downregulation of phosphorylated Akt (pAkt), reducing the viability and proliferative potential of tumor cells (Phillips et al., 2011).

Myricetin has demonstrated potent anti-cancer activity against pancreatic neoplasms by selectively inducing apoptosis in pancreatic cancer cells without affecting the viability of healthy pancreatic ductal cells. At the cellular level, myricetin treatment leads to significant apoptotic induction, as evidenced by increased Annexin V and TUNEL staining, along with upregulated expression of cleaved caspase-9 and cleaved caspase-3, which signifies robust activation of the intrinsic mitochondrial apoptotic cascade, reflecting a commitment to programmed cell death via cytochrome c-mediated signaling. *In vivo* studies using orthotopic mouse models implanted with MIA PaCa-2 and S2-013 pancreatic cancer cells further confirmed that myricetin treatment results in a marked reduction in tumor growth and metastasis (Phillips et al., 2011).

Despite myricetin’s strong anti-neoplastic properties, its limited systemic absorption and poor water solubility limit its translational relevance in clinical application. Its pharmacokinetic profile has been considerably enhanced by recent developments in drug administration, especially nanoparticle encapsulation techniques, which have increased both bioavailability and therapeutic efficacy (Javed et al., 2022). These results demonstrate myricetin’s therapeutic potential as a viable therapeutic candidate for focused intervention in pancreatic cancers.

#### 3.1.3 Kaempferol

Kaempferol is a naturally occurring flavonol that can be found in a wide range of foods, such as vegetables (kale, spinach, and broccoli), fruits (apples, grapes, and strawberries), and medicinal plants. According to (Calderon-Montano et al., 2011), kaempferol can also be found in traditional medicinal herbs, including Ginkgo biloba, Moringa oleifera, and Sophora japonica, as well as tea, especially green and black types. Because of its multifactorial qualities, such as its anti-inflammatory, anti-oxidant, anti-viral, anti-cancer, cardioprotective, and neuroprotective effects, kaempferol has attracted a lot of scientific attention. Interestingly, kaempferol has strong anti-cancer effects by carefully adjusting key oncogenic signaling pathways that control tumor growth, cellular transformation, and survival. Further research in the effect of kaempferol on Type 1 diabetes shows a significant reduction in blood glucose, triglyceride levels, an increase in high-density lipoprotein levels (HDL) and maintenance of the β-cell structure of the pancreas (Song et al., 2024). Also, it shows a protective effect against metabolic disorders that are caused by type 1 diabetes and thereby protects the pancreas from cellular damage (Song et al., 2024). Studies have shown that kaempferol has a protective role towards pancreatic β-cells from hyperglycaemia-induced apoptosis and dysfunction. Kaempferol has shown notable anti-diabetic efficacy by enhancing pancreatic β-cell viability and functional integrity, primarily through the induction of cyclic AMP (cAMP) signaling and the upregulation of key survival mediators such as Akt and Bcl-2 (Zhang and Liu, 2011).

Kaempferol is used extensively to treat primary inflammatory digestive diseases and other acute inflammations. Mitochondrial homeostasis is essential for multiple biological processes in the pancreas. Dysfunction can cause severe acute pancreatitis (SAP) characterised by inflammation, ROS generation, and impaired energy production, leading to pancreatic damage (Wen et al., 2024). Various studies have shown that kaempferol exhibits numerous therapeutic potentials in the suppression of mitochondrial impairment and anti-inflammation (Kim et al., 2018). Kaempferol loaded with ROS-responsive nanomaterials delivery system is effective in treating pancreatitis with maximum anti-oxidant and anti-inflammatory effects by improving mitochondrial fission and mitophagy (Wen et al., 2024).

Kaempferol exerts reactive oxygen species (ROS)-mediated anti-cancer effects by facilitating cell cycle arrest and apoptotic induction, while concurrently attenuating cellular proliferation and invasive potential, underscoring its capacity to disrupt tumorigenic progression through redox-sensitive signaling pathways. Also, kaempferol is likely to inhibit tumor growth by upregulation of ROS, thereby leading to the apoptosis of the tumor cells. Both *In vivo* and *In vitro* studies on pancreatic cancer cells showed that kaempferol induces ROS-dependent apoptosis by TGM2-mediated Akt/mTOR signaling pathways (Wang et al., 2021). Kaempferol extract from ginkgo biloba increased apoptosis of MIA PaCa-2 and PANC-1 cells in a dose-dependent manner. It exhibits broad-spectrum enzymatic inhibition, targeting key regulatory proteins including cAMP-phosphodiesterase, tyrosine kinases, cdc25 phosphatases, DNA topoisomerase II, topoisomerase I-mediated DNA ligation, and proline-directed protein kinases involved in fatty acid signaling. Its ability to alter a variety of cellular functions essential to signal transduction, proliferation, and genomic integrity is highlighted by this complex interference. Also, kaempferol shows an additive effect and can be used as a combination drug with gemcitabine or 5-FU for better therapeutic efficacy (Zhang et al., 2008). Tyrosine kinase receptor (EGFR) is activated when it interacts with the growth factor of EGF family. Also, overexpression and mutation of this receptor are observed in various cancers. Upregulation of this EGFR is seen in pancreatic cancer, which could be targeted by kaempferol (Lee and Kim, 2016). Molecular docking studies have revealed that kaempferol has a high binding affinity with EGFR (−8.9 kcal/mol). The concurrent administration of Kaempferol and erlotinib (ERL) elicits an anti-proliferative effect on PANC-1 and BxPC-3 pancreatic cancer cell lines and concomitantly enhances apoptotic induction under *in vitro* conditions (Zhang et al., 2021). Furthermore, functional annotation and pathway mapping implicate the PI3K/Akt axis and the mechanism underlying EGFR tyrosine kinase inhibitor (TKI) resistance as putative synergistic targets, reinforcing the therapeutic rationale for this dual-agent strategy in pancreatic carcinoma management (Zhang et al., 2021). Specifically, disrupting EGFR-associated signaling cascades by dose-dependent administration of kaempferol significantly reduced serum-stimulated migratory behaviour in MIA PaCa-2, PANC-1, and SNU-213 pancreatic cancer cell lines. This mechanistic interference underscores the potential of kaempferol to reduce metastatic capability by altering oncogenic receptor pathways (Lee and Kim, 2016).

#### 3.1.4 Morin

Morin (2′,3,4′,5,7-pentahydroxyflavone), a bioflavonoid found in moraceae plants, almonds, bilberries, and Cudrania tricuspidata bread leaves. Several investigations have shown its anti-inflammatory, antioxidant, anti-hypersensitive, hepatoprotective and anti-cancer activity in many cancers, which include breast, lung, liver, prostate, glioma, ovarian and tongue squamous carcinoma cells (Yao et al., 2017; Sithara et al., 2017; Ji et al., 2018; Lee et al., 2021). In PDAC, Morin shows a significant anti-tumor effect by inducing PINKI/Parkin-mediated mitophagy and mitochondrial function disruption. Also, morin leads to the modulation of epithelial-mesenchymal transition by suppressing its tumorigenic potential and cancer stemness (Sharma et al., 2026).

In addition, studies from our laboratory have highlighted the pronounced anti-cancer activity of morin in HCC and liver fibrosis models. In HCC, morin activated the Mst1/Hippo pathway, suppressing Wnt/β-catenin and canonical NF-κB pathways while also promoting apoptosis through the Mst1-JNK-caspase mechanism (Perumal et al., 2017b). Also, Morin inhibits hepatic stellate cell activation by regulating key signaling pathways, such as Hippo/YAP1 and TGF-β1/Smad signaling, thereby highlighting it as a versatile anti-fibrogenic agent (Perumal et al., 2017a).

### 3.2 Flavones

#### 3.2.1 Apigenin

Apigenin is a naturally occurring flavone found mostly in foods like parsley and the dried inflorescences of chamomile. Apigenin (4′,5,7-trihydroxyflavone) has gained significant attention in the field of oncology due to its bioactive properties (Lefort and Blay, 2013). Apigenin shows strong anti-proliferative effects on many cancer types, including pancreatic carcinoma, where it inhibits the growth by altering molecular pathways (Budhraja et al., 2012; Imran et al., 2020).

Pancreatic cancer is distinguished by its highly inflammatory tumor microenvironment that leads to poor prognosis. Interaction between Programmed death ligand 1 and 2 induces exhaustion and T cell energy, which paves the way for the invasion of immune cells and tumor progression (Villalobos-Ayala et al., 2020a). Studies have proven that the treatment of apigenin using murine pancreatic cancer cell lines such as panc02, UN-KPC960 and UN-KC-6141 leads to the alteration of expression of immune check-point molecules such as PD-L1, PD-L2, PD-1 and MHC-1. Further, apigenin mediated enhanced T cell proliferative responses, which led to the restoration of anti-tumor immunity (Villalobos-Ayala et al., 2020a). Src Homology 2 (SH2) domain-containing Inositol 5′-Phosphatase-1 (SHIP-1) is a master regulator and essential protein in myeloid development, function and tumor immunity. Studies show that the treatment with apigenin restores the SHIP-1 expression, which in turn increases the tumoricidal tumor-associated macrophages (TAMs) significantly, leading to the improved anti-tumor immune responses in TME in PDAC cancer models (Villalobos-Ayala et al., 2020b).

The activation of NF-κB abnormally, promotes neoplastic growth, which is the characteristic molecular event in the pathophysiology of pancreatic cancer (Prabhu et al., 2014). Sustained activation of NF-κB activation aids immune escape and plays a crucial role in chemoresistance, making it a central driver in pancreatic carcinogenesis. Convincing evidence demonstrates that apigenin effectively hinders NF-κB’s DNA-binding affinity and transcriptional activity within pancreatic cancer cells. This suppression is mediated through the activation of caspase-3 and subsequent PARP cleavage, culminating in apoptotic cell death (Wu et al., 2014). TNF-α-induced NF-κB activity, including DNA binding, transcription, IκB-α phosphorylation, p65 and p50 nuclear translocation, and IKK-β activity, is reduced when treated with apigenin (Malik et al., 2017). Also, apigenin decreases pancreatic cancer in nude mice, accompanied by the downregulation of IKK-β expression *in vivo*. Apigenin administration effectively suppresses IKK-β-driven activation of the NF-κB signaling axis, resulting in a marked attenuation of human pancreatic tumor cell proliferation across both *in vitro* and *in vivo* models (Wu et al., 2014).

Johnson and de Mejia (2013), have reported that the bioactive compound apigenin blocked the GSK-3/NF-κB signaling pathway, which causes the activation of the mitochondrial-related apoptosis pathway. Apigenin serves as a more effective inhibitor against GSK-3/NF-κB signaling in BxPC-3 and PANC-1 cells than the chemotherapeutic drug cisplatin and the GSK-3 inhibitor SB414286. In BxPC-3 pancreatic cancer cells, administration of apigenin elicited upregulation of 59 genes associated with cytokine and interleukin (including IL17A, IL17C, and IL17F) and lymphotoxin alpha (LTA) while decreasing the expression of 63 genes connected to inflammation and malignancy (Johnson and de Mejia, 2013). Apigenin mediates cell cycle arrest in BxPC-3 and PANC-1 pancreatic cancer cells by perturbing G2 phase progression, primarily through the downregulation of cyclin B1 expression. This disruption of mitotic entry underscores its role in impairing proliferative capacity via targeted modulation of cell cycle regulatory machinery. Apigenin with 5FU has shown a significant reduction in BxPC-3 cell growth than the individual effect (Johnson and Gonzalez de Mejia, 2013).

#### 3.2.2 Luteolin

Luteolin (3,4,5,7-tetrahydroxyflavone), a naturally occurring flavonoid, is ubiquitously distributed across a diverse array of dietary and medicinal plants, including peppers, thyme, celery, parsley, broccoli, rosemary, olive oil, oregano, cabbage, carrots, artichoke, chrysanthemum blossoms, and apple peels (Saleem et al., 2021). In the context of traditional Chinese medicine, luteolin-enriched phytotherapeutics have long been employed in the management of multifactorial disorders such as cancer and hypertension, attributed to their pleiotropic bioactivities, most notably anti-inflammatory, anti-allergic, and anti-cancer properties mediated via both anti-oxidant and pro-oxidant regulatory mechanisms (Saleem et al., 2021).

Recent studies have shown that luteolin decreased tumor growth such as breast, colorectal, pancreatic, lung, prostate, liver, skin, gastric and oral cancer models (Ganai et al., 2021). In pancreatic malignancy, luteolin has been shown to exert potent anti-tumor effects by triggering apoptotic cascades, enforcing cell cycle arrest, and attenuating oncogenic signaling networks.

In PDACs, luteolin (Lut) inhibited tumor formation and decreased the expression of dihydropyrimidine dehydrogenase (DPYD), an enzyme that breaks down pyrimidines like 5-fluorouracil (5-FU), which causes chemoresistance in PDAC. This is achieved by combining the 5-FU with luteolin, which has elevated the therapeutic potential in PDAC (Kato et al., 2021). In addition to preclinical models like BOP-induced tumors in hamsters, the reduction of the tumor size by luteolin treatment through the STAT3-DYPD pathway (Kato et al., 2021). Under *in vivo* conditions, luteolin has been shown to induce apoptosis in pancreatic cancer cells, notably BxPC-3, through targeted suppression of the KRAS/GSK-3β/NF-κB signaling axis. This molecular inhibition initiates mitochondrial cytochrome-C release, activates caspase-3, and diminishes nuclear localization of GSK-3β and NF-κB p65, collectively contributing to its pro-apoptotic and anti-tumor efficacy (Pandey et al., 2025). miR-301-3p, non-coding RNA plays an oncogenic role in PDAC, whereas treatment with luteolin downregulated miR-301-3p, leading to the anti-proliferative effects and apoptosis (MOENG et al., 2020). *In vivo* studies on PDAC animal models have shown that administration of luteolin (150 and 75 mg/kg) inhibited carcinogenesis via increasing amylase activity, reducing PDAC tumor burden, Ki-67 labelling index, pSTAT3 signal transduction (Rauf et al., 2024). Dihydropyridine dehydrogenase (DPYD) is upregulated in pancreatic cancer, that are associated with proliferation, invasion, metastasis and makes the cells resistant to 5-FU treatment. Also, the upregulation of DPYD in PDAC leads to the activation or upregulation of other genes that are involved in metallopeptidase activity, such as MMP9 and MEP1A (Kato et al., 2024) These upregulation genes by DPYD promote proliferation and angiogenesis by activating signaling pathways such as IL6R-IL6-STAT3. The significant involvement of DPYD-MMP9-MEP1A-STAT3 in PDAC has become a potential therapeutic target for PDAC. Luteolin inhibits the expression of DPYD, MEP1A and STAT3 signaling pathway in AsPC-1 cells that have high expression of MEP1A. The combined treatment of luteolin with 5-FU and gemcitabine inhibits PDAC by inhibiting the expression of DPYD. These studies conclude that combinatorial treatment of luteolin showed significant outcomes compared to standalone treatment (Kato et al., 2024).

### 3.3 Flavanones

#### 3.3.1 Hesperidin

Hesperidin, a natural flavonoid found widely in oranges, lemons, fruits and vegetables possesses anti-microbial, anti-cancer, antioxidant, anti-inflammatory and anti-diabetic properties. Also, hesperidin inhibits cell proliferation in various *in vitro* studies such as lungs, colon, liver, pancreas, ovarian, skin, and breast cancer cells. Hesperidin’s anti-cancer effects are mediated by controlling multicellular targets, regulating key molecular signaling pathways, and favouring tumorigenesis (Pandey and Khan, 2021). Hesperidin has been shown to significantly suppress tumour growth when used alone or in combination with chemotherapy medications such as doxorubicin, paclitaxel, tamoxifen, cyclophosphamide, temozolomide, pirarubicin and methotrexate. In pancreatic cancer cells, a combination of the bioactive flavonoid naringenin and hesperidin shows synergistic anti-cancer effects. In PANC-1 cells, 1–20 µM concentration of hesperidin showed potent anti-cancerous effects by downregulating p38 and FAK signaling pathways (Lee et al., 2019).

Lee et al. (2019) reported that fermented extract of Citrus unshiu peel (fCUP) contains hesperidin and naringenin, which exerts anti-cancer properties both *in vitro* and *in vivo* experimentation models. Cadmium accumulates in the organs such as the liver, pancreas and kidney, which are now considered as a potential target of its toxicity mediated through inflammation. Cadmium toxicity in the pancreas can lead to oxidative stress, inflammation, as well as apoptosis of pancreatic tissue, causing pancreatitis. Hesperidin protects cadmium-induced pancreatitis by inhibition of NF-κB, iNOS, TNF-α and IL-6 in the pancreas (Aja et al., 2020).

#### 3.3.2 Naringenin

Naringenin, a naturally occurring flavanone predominantly found in citrus fruits such as grapes, tomatoes, cherries possess anti-oxidant, anti-inflammatory and anti-cancer properties (Mir and Tiku, 2015). Moreover, Naringenin can arrest the cell cycle thereby inactivating the carcinogens in different cancers (Motallebi et al., 2022). In streptozotocin–nicotinamide induced diabetic rats, naringenin treatment either alone or in combination led to the restoration of pancreatic β-cell mass and enhanced glucose metabolism. It activates estrogen receptor β (ERβ), thereby improving insulin production in primary rat islets. In diabetic models, naringenin significantly improved glycemic control and preserved β-cell function, highlighting its potential as a therapeutic agent for diabetes management (Lin et al., 2023). When pancreatic cancer cell lines (AGS, HEK293, AsPC-1, PANC-1) are treated with different concentrations of naringenin leading to the suppression of invasion, EMT and inhibited TGF-β1-induced resistance to Gemcitabine (Lou et al., 2012). The multifunctional Prdx-1 has been investigated for its role in cell survival, proliferation, and apoptosis. The overexpression of the Prdx-1 has been reported in various cancers, including pancreatic cancer. Evaluating the therapeutic activity of naringenin in human pancreatic cancer cells (SNU-213) showed that naringenin induces apoptosis of the cancer cells through activating Prdx-1 inhibition pathway (Park et al., 2017). Recent data suggest that naringenin inhibits the TGF-β1/Smad3 signaling cascade, which in turn lowers the transcriptional and translational expression of EMT markers in AsPC-1 and PANC-1 pancreatic cancer cell lines. Interestingly, it also makes gemcitabine-resistant cells chemosensitive again, highlighting its potential as a therapeutic adjuvant to overcome drug resistance (Lou et al., 2012).

#### 3.3.3 Eriodictyol

Eriodictyol is a flavonoid, a subclass of flavanones. It can be found in dietary products such as citrus species and cereal grains, along with microbial *de novo* biosynthesis pathways. Eriodictyol exerts multifaceted regulatory effects across diverse cellular signaling pathways and has extensive therapeutic potential, including anti-oxidant, anti-inflammatory, anti-cancer, neuroprotective, cardioprotective, anti-diabetic, anti-obesity, and hepatoprotective activities (Islam et al., 2020). Recent studies have demonstrated that eriodictyol effectively induces apoptosis in SNU213 and PANC-1 pancreatic cancer cell lines. Mechanistically, the cytotoxic effect is mediated through the suppression of Akt and ERK phosphorylation, coupled with enhanced activation of the JNK signaling pathway contributing to eriodictyol-induced cell death (Oh et al., 2022). These findings underscore its potential as a promising anti-cancer candidate for therapeutic intervention in PDAC.

### 3.4 Flavanols

#### 3.4.1 Catechins

Catechins are flavanols, which are active compounds present in Camellia sinesis has anti-cancer, anti-microbial and anti-inflammatory properties. They are also used to treat cardiovascular diseases, neurological disorders, diabetes etc. Epicatechin (EC), Epigallocatechin (EGC), epicatechin-3-gallate (ECG), and epigallocatechin-3-gallate (EGCG) are important derivatives of catechins, out of which EGCG is the richest compound in green tea as catechins (Suhail et al., 2023; Suhail et al., 2019). Nuclear Factor-Kappa B (NF-κB), a key transcription factor that plays a vital role in various cancer progressions, including pancreatic cancer, liver, lung, and breast cancers (Suhail et al., 2021). Targeting or inhibiting the NF-κB signaling pathways using plant-based natural compounds could pave a cure towards NF-κB driven tumors. Molecular docking studies targeting NF-κB with green tea catechins derivatives have shown significant binding potential, thereby inhibiting its DNA-binding activity, eventually attenuating disease progression (Suhail et al., 2019).

Mutational activation of NF-κB has been strongly implicated in promoting malignant phenotypes in pancreatic cancer, including enhanced cellular proliferation, invasive potential, angiogenic signaling, and resistance to chemotherapeutic agents. Research has been done on catechin derivatives to analyse binding pose and molecular interaction with NF-κB computationally. Among the derivatives, EGCG binds to p65 subunit of NF-κB, thereby triggering apoptosis in pancreatic cancer cell lines (*In vitro* studies). EGCG also resulted in the reduction in the cell proliferation rate and downregulated the expression of c-Myc, MMP9 and MMP2 (Suhail et al., 2023). EGCG demonstrates potent anti-tumor activity in pancreatic cancer by concurrently inhibiting the ERK and PI3K/Akt signaling pathways while activating the transcription factor FKHRL1/FOXO3a. This dual regulatory mechanism contributes to the suppression of tumor growth, angiogenesis, and metastatic dissemination (Bimonte et al., 2015). Studies indicate that EGCG causes stress signals in MIA PaCa-2 pancreatic cancer cells by damaging mitochondria and activating ROS-mediated JNK pathways (Qanungo et al., 2005).

Combined therapy of catechins and their derivatives with other phytocompounds or drugs shows a synergistic effect, reduced side effects, and enhanced anti-cancer activity. Research done to evaluate the potential of catechins on electrochemotherapy (ECT) with cisplatin, where an *in vitro* study showed increased efficacy of cisplatin with decreased viability of human pancreatic cancer cells (Michel et al., 2018). Pterostibene, a naturally occurring stilbenoid that is present in abundance in blueberries, possesses all the therapeutic potential of catechins. When employed as a combinatorial therapeutic strategy, the agents exhibit marked anti-proliferative effects on MIA PaCa-2 and PANC-1 in a temporally regulated and dose-responsive manner. Moreover, S-phase arrest favoring mitochondrial derived apoptotic pathway was observed in MIA PaCa-2 cells with the combined treatment of these Phytochemical compounds (Kostin et al., 2012). Like pterostibene, a combination of bleomycin (BLM) with EGCG also shows anti-proliferative effect leading to the apoptosis of MIA PaCa-2 cells (Bimonte et al., 2015). Also, studies comparing the effects of epicatechin gallate and catechin gallate with EGCG in PDAC cells (PancTu-1, PANC-1, Panc89 and BxPC3 cells) were conducted. The results show positive for all the catechins by inhibiting the cell proliferation rate, but the effects of epicatechin gallate and catechin gallate were much stronger than EGCG (Kürbitz et al., 2011). Network pharmacological studies of the hub genes in pancreatic cancer against the combined therapy of kaempferol and catechins show strong binding affinity, thereby showing a promising path for the formulation of an anti-pancreatic cancer drug. Also, the combination of the catechins and inositol hexa phosphate (IL6) shows a strong anti-proliferative effect in pancreatic cancer cells, suggesting that these combined therapies can be used for better treatment strategies with fewer side effects (McMillan et al., 2007).

#### 3.4.2 Theaflavins

Theaflavins also belong to flavanols that have their source in black tea, elucidating anti-cancer effects in various cancer cells (Lahiry et al., 2008). Theaflavins promote apoptosis in p53-mutated breast cancer cells; therapeutic intervention activates the Fas death receptor–caspase-8 apoptotic pathway while concurrently inhibiting the pAkt/pBad cell survival axis. The convergence of these distinct signaling cascades culminates in mitochondrial dysfunction, evidenced by the loss of cytochrome C release, mitochondrial transmembrane potential (MTP), and subsequent activation of caspase-9 and caspase-3, ultimately driving apoptotic cell death (Lahiry et al., 2008). Very few works on theaflavins in breast cancer and inhibition of pancreatic lipase were reported (Glisan et al., 2017).

### 3.5 Isoflavones

#### 3.5.1 Genistein

It is an important Isoflavone among all the available ones because of its numerous pharmacological potentials and is not limited to specific cancer types (Liang et al., 2024). STAT3 signaling pathway plays a major role in many cancer types, where the STAT protein is involved in stimulating and maintaining the proliferative, anti-apoptotic, and inflammatory pathways of the tumor cells. Research studies on the effect of genistein in MIA PaCa-2 cells inhibited the phosphorylation of STAT3 protein in a dose-dependent manner. Genistein, a plant-derived bioactive isoflavone predominantly found in soybeans, exhibits potent anti-cancer activity against pancreatic malignancies by inducing apoptotic cell death. Additionally, it enhances chemosensitivity through targeted inhibition of key survival pathways, including PI3K/Akt, and NF-κB, thereby disrupting cellular resistance mechanisms. Investigations into combinatorial therapeutic strategies have revealed that genistein potentiates the efficacy of erlotinib and gemcitabine in pancreatic cancer cell lines by suppressing EGFR-independent activation of the Akt/NF-κB signaling axis. This synergistic interaction effectively lowers the apoptotic threshold, thereby enhancing cell susceptibility to programmed death and offering a promising approach to overcoming chemoresistance (El-Rayes et al., 2006).

Both *in vitro* and *in vivo* experimental models have substantiated the anti-tumor efficacy of 5-FU and Genistein on pancreatic cancer is high. AXP107-11, a crystalline form of genistein, has the potential to sensitize the tumor cells to chemotherapy. In PDAC cells, the AXP107-11 was used in combination with gemcitabine and alone, both *in vitro* and *in vivo,* to determine the efficacy of the drugs. The results concluded that the combined therapy has a synergistic chemo-enhancing effect, leading to the identification of 41 gene signatures that may differentiate between responsive tumors (Löhr et al., 2016). Keeping aside the combination of genistein with the available drug, a new approach to the treatment of genistein with miRNA was also developed. The combination of miR-223 inhibitor with genistein has led to the reversal of EMT compared with the standalone treatment. Furthermore, inhibition of migration and invasion while increasing the gemcitabine sensitivity in pancreatic cancer cells was also observed (Ma et al., 2016). Also, studies showed that Genistein modulates tumor growth by interfering with glucose-derived carbon flux into nucleic acid ribose through the nonoxidative arm of the pentose phosphate pathway. This metabolic reprogramming disrupts nucleotide biosynthesis, thereby impairing proliferative capacity in pancreatic cancer cells. It affects the glucose utilization pathways of the tumor cells and inhibits its proliferation. Curcumin and genistein exert multifaceted anti-tumor effects by downregulating key oncogenic mediators, including NF-κB, COX-2, VEGF, miRNA-22, Bcl-xL, and Bcl-2 that are critically involved in pancreatic tumor initiation, cellular proliferation, aggressive phenotypic transformation, and metastatic dissemination. Concurrently, they enhance the expression of pro-apoptotic proteins such as Bax and caspases, thereby promoting programmed cell death and suppressing tumor viability, both *in vitro* and *in vivo* models (Dariya et al., 2019).

#### 3.5.2 Diadzein

Like genistein, daidzein is an isoflavone that is present in high concentration in soybeans and legumes. Daidzein-rich foods are used to treat various diseases, including cancer. It’s a natural phytoestrogen that is used mainly for the treatment of breast cancer (Singla et al., 2024). It exhibits a broad spectrum of therapeutic properties, encompassing anti-cancer efficacy, particularly against hormone-responsive breast malignancies, as well as immunomodulatory, anti-inflammatory, cardiovascular-supportive, and glycemic-regulating effects and neuroprotective activities (Singla et al., 2024). Daidzein causes death in MCF-7 cells through the mitochondrial route, reducing the ER α/β ratio and increasing the intracellular ROS levels (Kumar and Chauhan, 2021). Daidzein has shown better promise against breast cancer, but its effects on pancreatic cancer remain unexplored.

#### 3.5.3 Anthocyanins

Polyphenols, particularly anthocyanins (ACNs), are potent bioactive compounds capable of scavenging oxygenated free radicals, inhibiting or activating enzymes, and functioning as metal chelators to prevent damage to membrane lipids, proteins, and nucleic acids. Comprising up to 80% of total polyphenolic pigments in fruits and vegetables, water-soluble flavonoid pigment ACNs have anti-inflammatory, anti-angiogenesis, antioxidant, and anti-proliferative properties, hence quite versatile and flexible in medical applications. Despite their low bioavailability, large amounts of dietary ACNs are converted by gut bacteria into phenolic acids and other bioactive metabolites, therefore greatly enhancing their therapeutic potential (Gašić et al., 2020; Kuntz et al., 2017).

The low physiological concentration of ACNs has demonstrated growth-inhibitory effects on pancreatic cancer cell lines like BxPC-3, reduced cell adhesion and migration in various other cancer cell lines, including HCT-116 and MDA-MB-231. The cell death involves triggering the caspase signaling pathway, initiating PARP cleavage, disrupting mitochondrial membrane integrity, and facilitating cytochrome C release into the cytosol (Hui et al., 2010). Additionally, ACNs suppress oncogenic proteins such as FOXM1 and NF-κB in cholangiocarcinoma, further supporting their anti-cancer role. ACN metabolite a new cancer preventive agent because of their anti-migratory effects on PANC-1 cells, which have also been linked to decreased NF-κB p65 and FAK phosphorylation as well as decreased ROS levels. This suppresses important intracellular signaling pathways associated with cancer cell migration and cancer metastasis ultimately (Kuntz et al., 2017; Mostafa et al., 2023). A combination of ACNs with gemcitabine have shown to overcome drug resistance and achieve the synergistic anti-cancer effects, despite gemcitabine’s chemoresistance limits (Koltai et al., 2022). ACNs enhance the cytotoxic effect of gemcitabine drastically in the gemcitabine-resistant KKU214GemR cell line, underscoring their function in making resistant cancer cells more susceptible to the conventional treatment (Intuyod et al., 2018). A randomized trial conducted with the intervention of anthocyanins-rich juices given to healthy subjects, and their plasma metabolites were isolated. The plasma metabolites treated with PANC-1 cancer cells show decreased migration and expression of cell adhesion molecules through NF-κB and FAK pathways (Mostafa et al., 2023). A similar study by Kuntz et al. (2017), Kuntz et al. (2017) also shows reduced tumor cell migration, minimized ROS levels and diminished mRNA levels of NF-κB, MMP-2, and MMP-9. Together, these findings highlight the potential of ACNs as innovative agents in the prevention and treatment of cancer, either alone or in conjunction with currently available chemotherapies like gemcitabine, offering a comprehensive strategy to address chemoresistance in PDAC.

##### 3.5.3.1 Cyanidin

Cyanidin, a notable anthocyanin, has been widely found in berries, cherries and black rice. This cyanidin has a vast range of bioactivities such as anti-oxidant, anti-diabetic, anti-inflammatory, and notable anti-cancer properties. Though the bioavailability of the cyanidin is limited, gut microbiota fermentation significantly enhances the therapeutic efficacy of cyanidin thereby altering the cellular pathway and scavenging of ROS. Delphinidin, cyanidin, petunidin, malvidin, pelargonidin and peonidin-3-glucoside are the predominant polyphenols found to have potent anti-cancer activities in various cancers including PDAC (Farhan et al., 2022).

Cyanidin effectively suppresses the activity of α-glucosidase in the intestinal epithelium and α-amylase secreted by the pancreas, both of which play central roles in the enzymatic breakdown of dietary carbohydrates (Akkarachiyasit et al., 2010). In a diabetic mouse model, cyanidin preserved pancreatic islet architecture and stimulated insulin secretion, decelerating disease progression. Cyanidin has demonstrated significant anti-cancer activity by dose-dependent inhibition of NF-κB-mediated MMP-2 production and decreased cellular adherence to type I collagen in highly metastatic A549 lung cancer cells (Chen et al., 2006). According to Safdar et al. (2023) Cyanidin works mechanistically by suppressing RAS and MAPK signaling and stimulating caspase-3 and p38 MAPK activity. This will cause cell cycle arrest, cellular differentiation, and redox homeostasis modulation.

Mulberry fruit-derived cyanidin-3-glucoside (C3G) shows cytoprotective effects against H2O2-mediated oxidative stress-induced pancreatic β-cell apoptosis by lowering lipid peroxidation and controlling apoptotic signaling pathways in MIN6N β-cells, which in turn decreased the apoptotic rate (Lee J. S. et al., 2015). The ROS scavenging and MG-trapping action of cyanidin, which shields DNA from oxidative damage and protein glycation, was further highlighted by the fact that it successfully preserved cell viability and decreased apoptosis brought on by methylglyoxal (MG) exposure (Suantawee et al., 2020). Cyanidin exerts its anti-cancer potential by modifying proteolytic enzymes like MMP-2 and u-PA and simultaneously raising the levels of their endogenous suppressors TIMP-2 and PAI in a dose-dependent manner (Chen et al., 2006). Collectively, cyanidin’s immense biological functions, mainly its anti-oxidative and anti-cancer effects, make it a reliable candidate for therapeutic applications across various diseases. However, its anti-cancer mechanism in PDAC is not well explored.

## 4 Clinical cohort findings

The clinical trials play a major role in the deep understanding of the mechanisms of flavonoids with respect to safety and efficacy (Ding and Yu, 2025). A clinical cohort study of 326 patients with pancreatic cancer and 652 healthy patient controls was conducted. FFQ analysis revealed that there is a negative relationship between the intake of flavonoids, such as proanthocyanidins, and the risk of developing pancreatic cancer (Rossi et al., 2012). Adding to the above study, eating fruits with proanthocyanidins has also been clinically shown to reduce the risk of developing pancreatic cancer (Rossi et al., 2012). A Cohort study including diverse ethnic groups analyzed the efficacy of quercetin, kaempferol, and myricetin in pancreatic cancer, revealed reduced risk of developing tumors, especially in smokers (Nöthlings et al., 2007). The phase Ib clinical study in pancreatic cancer evaluating AXP107-11, a novel crystalline form of genistein, has shown a favorable pharmacokinetic profile when used in combination with gemcitabine, enhancing bioavailability without hematological or non-hematological toxicity. This novel drug formulation has enhanced the therapeutic potential of genistein with reduced side effects compared to its native form, and it is beneficial for elderly patients (Löhr et al., 2016).

In other cancers, a clinical study evaluating the efficacy of apigenin in colorectal cancer in combination with EGCG showed that patients receiving traditional treatments have shown a reduced tumor recurrence rate (7%) in the flavonoids-treated group, whereas the non-apigenin treatment groups showed a recurrence rate of 47%. These findings suggest that the long-term treatment of flavonoids could reduce the tumor recurrence rate in patients with colorectal cancer (Hoensch et al., 2008). A similar two-cohort follow-up study showed that the patients with increased intake of flavonols reduced the mortality rate in patients with colorectal cancer (Shi et al., 2023). A clinical study in the Shanghai cohort of women investigating the relationship between dietary and urinary isoflavonoid levels with respect to liver cancer revealed that the higher the urinary concentration of genistein, lowers the risk of developing liver cancer. Also, the results from this clinical trial support the hypothesis that exposure to isoflavonoids, specifically genistein, may lead to cancer prevention, mainly liver cancer (Zhang et al., 2019).

## 5 Current challenges and future prospects

Despite the rising evidence indicating the anti-cancer potential of plant-derived flavonoids in pancreatic ductal adenocarcinoma (PDAC), several challenges still limit their clinical translation. Most flavonoid-related studies have been limited to *in vitro* and *in vivo* experimental models, while well-designed clinical trials to assess their efficacy, safety, pharmacokinetics, and long-term effects in PDAC patients remain scarce. Moreover, many flavonoids exhibit low water solubility, limited bioavailability, rapid metabolism, and less tumor-specific accumulation, substantially reducing their anti-cancer potential in *in vivo* models (Park and M. Pezzuto, 2012). Furthermore, the current clinical understanding of the molecular signaling pathway regulation mediated by flavonoids exhibiting anti-cancer efficacy are limited with respect to their interactions with tumor microenvironment, cancer stem cells, immune cells, and desmoplastic stroma, and therapeutic resistance. Muhammad et al. (2022) stated that most of the natural drugs exert multi-targeted effects on different signaling pathways, raising concerns about the identification of the primary molecular targets and patient-specific therapeutic responses. Additionally, differences in flavonoid sources, methods of extraction, formulation strategies, and experimental designs hinder the reproducibility and comparison of results between studies.

Future research should be aimed at integrated multi-omics approaches, including genomics, transcriptomics, proteomics, and metabolomics, to extensively understand flavonoid-regulated molecular interactions and therapeutic responses. In addition, use of clinically relevant platforms such as patient-derived organoids, patient-derived xenografts, and genetically engineered mouse models may improve the translational relevance of efficacy studies. Furthermore, the novel generation of nanotechnology-based delivery systems, such as nanoparticles, liposomes, and ligand-targeted formulations, might increase the bioavailability, stability, and tumor-selective concentrations of flavonoids, thereby increasing their therapeutic effects (Dewanjee et al., 2023). Additional studies investigating flavonoids in combination with conventional chemotherapies, immunotherapies, and targeted agents may provide effective precision-based therapeutic strategies for overcoming drug resistance and improving clinical outcomes (Muhammad et al., 2022; Naeem et al., 2022).

## 6 Conclusion

Flavonoids belong to the varied class of phytochemicals abundantly found in plant-based sources (vegetables, fruits) and in commonly consumed beverages (coffee, tea, and cocoa). Research has shown that regular intake or consumption of flavonoids helps to maintain good health and to reduce life-threatening diseases like cancer. Also, the commercial availability of flavonoids and their phytochemical properties, such as anti-cancer, anti-diabetic, and anti-inflammatory, have made flavonoids a better option for treating different types of cancer. The preclinical studies of different types of flavonoids on pancreatic cancer have shown good therapeutic effects, reducing the load of chemotherapeutic drugs, thereby reducing the adverse side effects, as well as increasing the overall survival rate. The combinatorial treatment of flavonoids with chemotherapeutic drugs for PDAC shows promising results, paving a new perspective for PDAC treatment worldwide. Despite the vast preclinical evidence supporting the therapeutic potential of flavonoids in PDAC, there is only a limited number of clinical trials evaluating their efficacy. To date, only a few early clinical studies on flavonoids, particularly genistein, have been conducted in PDAC patients. Currently, the mechanistic understanding of flavonoids in PDAC therapeutics is mainly from *in vitro* and preclinical *in vivo* studies, thus emphasizing the notable gap between experimental and clinical translational research. Therefore, this review also highlights the urgent need for well-designed, large-scale clinical trials to validate the preclinical findings of flavonoids for effective tumor management and better clinical outcomes of PDAC patients.

## References

[B1] Adamska A. Domenichini A. Falasca M. (2017). Pancreatic ductal adenocarcinoma: current and evolving therapies. Int. J. Mol. Sci. 18, 1338. 10.3390/ijms18071338 28640192 PMC5535831

[B2] Aja P. M. Izekwe F. I. Famurewa A. C. Ekpono E. U. Nwite F. E. Igwenyi I. O. (2020). Hesperidin protects against cadmium-induced pancreatitis by modulating insulin secretion, redox imbalance and iNOS/NF-ĸB signaling in rats. Life Sci. 259, 118268. 10.1016/j.lfs.2020.118268 32800830

[B3] Akkarachiyasit S. Charoenlertkul P. Yibchok-anun S. Adisakwattana S. (2010). Inhibitory activities of cyanidin and its glycosides and synergistic effect with acarbose against intestinal α-Glucosidase and pancreatic α-Amylase. Int. J. Mol. Sci. 11, 3387–3396. 10.3390/ijms11093387 20957102 PMC2956102

[B4] Almatroodi S. A. Rahmani A. H. (2025). Unlocking the pharmacological potential of myricetin against various pathogenesis. Int. J. Mol. Sci. 26, 4188. 10.3390/ijms26094188 40362425 PMC12071824

[B5] Angst E. Park J. L. Moro A. Lu Q.-Y. Lu X. Li G. (2013). The flavonoid quercetin inhibits pancreatic cancer growth *in vitro* and *in vivo* . Pancreas 42, 223–229. 10.1097/MPA.0b013e318264ccae 23000892 PMC3530623

[B6] Appari M. Babu K. R. Kaczorowski A. Gros W. Her I. (2014). Sulforaphane, quercetin and catechins complement each other in elimination of advanced pancreatic cancer by miR-let-7 induction and K-ras inhibition. Int. J. Oncol. 45, 1391–1400. 10.3892/ijo.2014.2539 25017900 PMC4151818

[B7] Arnold M. Abnet C. C. Neale R. E. Vignat J. Giovannucci E. L. McGlynn K. A. (2020). Global burden of 5 major types of gastrointestinal cancer. Gastroenterology 159, 335–349.e15. 10.1053/j.gastro.2020.02.068 32247694 PMC8630546

[B8] Bao B. Wang Z. Ali S. Kong D. Banerjee S. Ahmad A. (2011). Over expression of FoxM1 leads to epithelial–mesenchymal transition and cancer stem cell phenotype in pancreatic cancer cells. J. Cell. Biochem. 112, 2296–2306. 10.1002/jcb.23150 21503965 PMC3155646

[B9] Baqer S. H. Al-Shawi S. G. Al-Younis Z. K. (2024). Quercetin, the potential powerful flavonoid for human and food: a review. Front. Biosci. 16, 30. 10.31083/j.fbe1603030 39344383

[B10] Bhatia R. Siddiqui J. A. Ganguly K. Thompson C. M. Cannon A. Aithal A. (2023). Muc4 loss mitigates epidermal growth factor receptor activity essential for PDAC tumorigenesis. Oncogene 42, 759–770. 10.1038/s41388-022-02587-1 36624189 PMC10198580

[B11] Bimonte S. Leongito M. Barbieri A. Del Vecchio V. Barbieri M. Albino V. (2015). Inhibitory effect of (−)-epigallocatechin-3-gallate and bleomycin on human pancreatic cancer MiaPaca-2 cell growth. Infect. Agent. Cancer 10, 22. 10.1186/s13027-015-0016-y 26225138 PMC4518601

[B12] Budhraja A. Gao N. Zhang Z. Son Y.-O. Cheng S. Wang X. (2012). Apigenin induces apoptosis in human leukemia cells and exhibits anti-leukemic activity *in vivo* . Mol. Cancer Ther. 11, 132–142. 10.1158/1535-7163.MCT-11-0343 22084167 PMC4430727

[B13] Calderon-Montano M. Burgos-Moron E. Perez-Guerrero C. Lopez-Lazaro M. (2011). A review on the dietary flavonoid kaempferol. Mini-Reviews Med. Chem. 11, 298–344. 10.2174/138955711795305335 21428901

[B14] Cao C. Sun L. Mo W. Sun L. Luo J. Yang Z. (2015). Quercetin mediates β-Catenin in pancreatic cancer stem-like cells. Pancreas 44, 1334–1339. 10.1097/MPA.0000000000000400 26284537

[B15] Casarcia N. Rogers P. Guld E. Iyer S. Li Y. Burcher J. T. (2025). Phytochemicals for the prevention and treatment of pancreatic cancer: current progress and future prospects. Br. J. Pharmacol. 182, 2181–2234. 10.1111/bph.16249 37740585

[B16] Chen P.-N. Chu S.-C. Chiou H.-L. Kuo W.-H. Chiang C.-L. Hsieh Y.-S. (2006). Mulberry anthocyanins, cyanidin 3-rutinoside and cyanidin 3-glucoside, exhibited an inhibitory effect on the migration and invasion of a human lung cancer cell line. Cancer Lett. 235, 248–259. 10.1016/j.canlet.2005.04.033 15975709

[B17] Chugh S. Barkeer S. Rachagani S. Nimmakayala R. K. Perumal N. Pothuraju R. (2018). Disruption of C1galt1 gene promotes development and metastasis of pancreatic adenocarcinomas in mice. Gastroenterology 155, 1608–1624. 10.1053/j.gastro.2018.08.007 30086262 PMC6219903

[B19] Dariya B. Govardhanagiri S. Rajitha B. Aliya S. Alam A. Nagaraju G. P. (2019). “Curcumin and genistein enhance the sensitivity of pancreatic cancer to chemotherapy,” in Breaking Tolerance to Pancreatic Cancer Unresponsiveness to Chemotherapy (Elsevier), 87–109. 10.1016/B978-0-12-817661-0.00006-8

[B20] Davoodvandi A. Shabani Varkani M. Clark C. C. T. Jafarnejad S. (2020). Quercetin as an anticancer agent: focus on esophageal cancer. J. Food Biochem. 44, e13374. 10.1111/jfbc.13374 32686158

[B21] Dewanjee S. Chakraborty P. Bhattacharya H. Singh S. K. Dua K. Dey A. (2023). Recent advances in flavonoid-based nanocarriers as an emerging drug delivery approach for cancer chemotherapy. Drug Discov. Today 28, 103409. 10.1016/j.drudis.2022.103409 36265733

[B22] Ding Y. Yu Y. (2025). Therapeutic potential of flavonoids in gastrointestinal cancer: focus on signaling pathways and improvement strategies. Mol. Med. Rep. 31, 109. (Review). 10.3892/mmr.2025.13474 40017144 PMC11884236

[B23] Du Z. Büttner U. F. G. Wirth H. L. Gerstenlauer M. Manfras U. Loidl R. (2026). NF-κB inducing kinase (NIK) deletion accelerates KRAS-driven pancreatic cancer in association with tumor microenvironment remodeling. Cell Death Dis. 17, 513. 10.1038/s41419-026-08877-w 42203767 PMC13216544

[B24] El-Rayes B. F. Ali S. Ali I. F. Philip P. A. Abbruzzese J. Sarkar F. H. (2006). Potentiation of the effect of erlotinib by genistein in pancreatic cancer: the role of akt and nuclear Factor-κB. Cancer Res. 66, 10553–10559. 10.1158/0008-5472.CAN-06-2333 17079479

[B25] Falconi M. (2021). Total pancreatectomy: how, when and why? Updat. Surg. 73, 1203–1204. 10.1007/s13304-021-01134-z 34286418

[B26] Farhan M. Rizvi A. Ali F. Ahmad A. Aatif M. Malik A. (2022). Pomegranate juice anthocyanidins induce cell death in human cancer cells by mobilizing intracellular copper ions and producing reactive oxygen species. Front. Oncol. 12, 998346. 10.3389/fonc.2022.998346 36147917 PMC9487716

[B27] Farhan M. Rizvi A. Aatif M. Ahmad A. (2023). Current understanding of flavonoids in cancer therapy and prevention. Metabolites 13, 481. 10.3390/metabo13040481 37110140 PMC10142845

[B28] Feng Y.-B. Chen L. Chen F.-X. Yang Y. Chen G.-H. Zhou Z.-H. (2023). Immunopotentiation effects of apigenin on NK cell proliferation and killing pancreatic cancer cells. Int. J. Immunopathol. Pharmacol. 37, 3946320231161174. 10.1177/03946320231161174 36848930 PMC9974612

[B29] Gaidhani R. H. Balasubramaniam G. (2021). An epidemiological review of pancreatic cancer with special reference to India. Indian J. Med. Sci. 73, 99–109. 10.25259/IJMS_92_2020

[B30] Ganai S. A. Sheikh F. A. Baba Z. A. Mir M. A. Mantoo M. A. Yatoo M. A. (2021). Anticancer activity of the plant flavonoid luteolin against preclinical models of various cancers and insights on different signalling mechanisms modulated. Phyther. Res. 35, 3509–3532. 10.1002/ptr.7044 33580629

[B31] Gašić U. Ćirić I. Pejčić T. Radenković D. Djordjević V. Radulović S. (2020). Polyphenols as possible agents for pancreatic diseases. Antioxidants 9, 547. 10.3390/antiox9060547 32585831 PMC7346180

[B32] Glisan S. L. Grove K. A. Yennawar N. H. Lambert J. D. (2017). Inhibition of pancreatic lipase by Black tea theaflavins: comparative enzymology and *in silico* modeling studies. Food Chem. 216, 296–300. 10.1016/j.foodchem.2016.08.052 27596423 PMC5228287

[B33] Guo Y. Tong Y. Zhu H. Xiao Y. Guo H. Shang L. (2021). Quercetin suppresses pancreatic ductal adenocarcinoma progression via inhibition of SHH and TGF-β/Smad signaling pathways. Cell Biol. Toxicol. 37, 479–496. 10.1007/s10565-020-09562-0 33070227

[B34] Haugvik S.-P. Janson E. T. Österlund P. Langer S. W. Falk R. S. Labori K. J. (2016). Surgical treatment as a principle for patients with high-grade pancreatic neuroendocrine carcinoma: a nordic multicenter comparative study. Ann. Surg. Oncol. 23, 1721–1728. 10.1245/s10434-015-5013-2 26678407

[B35] Helen H. Gunawan M. C. Halim P. Dinata M. R. Ahmed A. Dalimunthe A. (2024). Flavonoids as modulators of miRNA expression in pancreatic cancer: pathways, mechanisms, and therapeutic potential. Biomed. Pharmacother. 179, 117347. 10.1016/j.biopha.2024.117347 39241569

[B36] Hoensch H. Groh B. Edler L. Kirch W. (2008). Prospective cohort comparison of flavonoid treatment in patients with resected colorectal cancer to prevent recurrence. World J. Gastroenterol. 14, 2187–2193. 10.3748/wjg.14.2187 18407592 PMC2703843

[B37] Hu J.-X. Zhao C.-F. Chen W.-B. Liu Q.-C. Li Q.-W. Lin Y.-Y. (2021). Pancreatic cancer: a review of epidemiology, trend, and risk factors. World J. Gastroenterol. 27, 4298–4321. 10.3748/wjg.v27.i27.4298 34366606 PMC8316912

[B39] Hui C. Bin Y. Xiaoping Y. Long Y. Chunye C. Mantian M. (2010). Anticancer activities of an anthocyanin-rich extract from black rice against breast cancer cells *in vitro* and *in vivo* . Nutr. Cancer 62, 1128–1136. 10.1080/01635581.2010.494821 21058201

[B40] Imran M. Aslam Gondal T. Atif M. Shahbaz M. Batool Qaisarani T. Hanif Mughal M. (2020). Apigenin as an anticancer agent. Phyther. Res. 34, 1812–1828. 10.1002/ptr.6647 32059077

[B41] Intuyod K. Priprem A. Pairojkul C. Hahnvajanawong C. Vaeteewoottacharn K. Pinlaor P. (2018). Anthocyanin complex exerts anti-cholangiocarcinoma activities and improves the efficacy of drug treatment in a gemcitabine-resistant cell line. Int. J. Oncol. 52, 1715–1726. 10.3892/ijo.2018.4306 29512768

[B42] Islam A. Islam M. S. Rahman M. K. Uddin M. N. Akanda M. R. (2020). The pharmacological and biological roles of eriodictyol. Arch. Pharm. Res. 43, 582–592. 10.1007/s12272-020-01243-0 32594426

[B44] Javed Z. Khan K. Herrera-Bravo J. Naeem S. Iqbal M. J. Raza Q. (2022). Myricetin: targeting signaling networks in cancer and its implication in chemotherapy. Cancer Cell Int. 22, 239. 10.1186/s12935-022-02663-2 35902860 PMC9336020

[B45] Ji Y. Jia L. Zhang Y. Xing Y. Wu X. Zhao B. (2018). Antitumor activity of the plant extract morin in tongue squamous cell carcinoma cells. Oncol. Rep. 40, 3024–3032. 10.3892/or.2018.6650 30132559

[B47] Johansson H. Andersson R. Bauden M. Hammes S. Holdenrieder S. Ansari D. (2016). Immune checkpoint therapy for pancreatic cancer. World J. Gastroenterol. 22, 9457–9476. 10.3748/wjg.v22.i43.9457 27920468 PMC5116591

[B48] Johnson J. L. de Mejia E. G. (2013). Flavonoid apigenin modified gene expression associated with inflammation and cancer and induced apoptosis in human pancreatic cancer cells through inhibition of GSK ‐3β/NF ‐κ B signaling Cascade. Mol. Nutr. Food Res. 57, 2112–2127. 10.1002/mnfr.201300307 23943362

[B49] Johnson J. L. Gonzalez de Mejia E. (2013). Interactions between dietary flavonoids apigenin or luteolin and chemotherapeutic drugs to potentiate anti-proliferative effect on human pancreatic cancer cells, *in vitro* . Food Chem. Toxicol. 60, 83–91. 10.1016/j.fct.2013.07.036 23871783

[B50] Johnson J. L. Dia V. P. Wallig M. Gonzalez de Mejia E. (2015). Luteolin and gemcitabine protect against pancreatic cancer in an orthotopic mouse model. Pancreas 44, 144–151. 10.1097/MPA.0000000000000215 25237909

[B52] Kato H. Naiki-Ito A. Suzuki S. Inaguma S. Komura M. Nakao K. (2021). DPYD, down-regulated by the potentially chemopreventive agent luteolin, interacts with STAT3 in pancreatic cancer. Carcinogenesis 42, 940–950. 10.1093/carcin/bgab017 33640964 PMC8283735

[B53] Kato H. Sato M. Naiki‐Ito A. Inaguma S. Sano M. Komura M. (2024). The role of DPYD and the effects of DPYD suppressor luteolin combined with 5‐ FU in pancreatic cancer. Cancer Med. 13, e70124. 10.1002/cam4.70124 39158384 PMC11331593

[B54] Kim T. W. Lee S. Y. Kim M. Cheon C. Ko S.-G. (2018). Kaempferol induces autophagic cell death via IRE1-JNK-CHOP pathway and inhibition of G9a in gastric cancer cells. Cell Death Dis. 9, 875. 10.1038/s41419-018-0930-1 30158521 PMC6115440

[B55] Koltai T. Reshkin S. J. Carvalho T. M. A. Di Molfetta D. Greco M. R. Alfarouk K. O. (2022). Resistance to gemcitabine in pancreatic ductal adenocarcinoma: a physiopathologic and pharmacologic review. Cancers (Basel) 14, 2486. 10.3390/cancers14102486 35626089 PMC9139729

[B56] Kostin S. F. McDonald D. E. McFadden D. W. (2012). Inhibitory effects of (-)-epigallocatechin-3-gallate and pterostilbene on pancreatic cancer growth *in vitro* . J. Surg. Res. 177, 255–262. 10.1016/j.jss.2012.04.023 22583593

[B57] Kubatka P. Mazurakova A. Samec M. Koklesova L. Zhai K. AL-Ishaq R. (2021). Flavonoids against non-physiologic inflammation attributed to cancer initiation, development, and progression—3PM pathways. EPMA J. 12, 559–587. 10.1007/s13167-021-00257-y 34950252 PMC8648878

[B58] Kumar V. Chauhan S. (2021). Daidzein induces intrinsic pathway of apoptosis along with ER α/β ratio alteration and ROS production. Asian Pac. J. Cancer Prev. 22, 603–610. 10.31557/APJCP.2021.22.2.603 33639680 PMC8190374

[B59] Kuntz S. Kunz C. Rudloff S. (2017). Inhibition of pancreatic cancer cell migration by plasma anthocyanins isolated from healthy volunteers receiving an anthocyanin-rich berry juice. Eur. J. Nutr. 56, 203–214. 10.1007/s00394-015-1070-3 26476633

[B60] Kürbitz C. Heise D. Redmer T. Goumas F. Arlt A. Lemke J. (2011). Epicatechin gallate and catechin gallate are superior to epigallocatechin gallate in growth suppression and anti‐inflammatory activities in pancreatic tumor cells. Cancer Sci. 102, 728–734. 10.1111/j.1349-7006.2011.01870.x 21241417

[B61] Lahiry L. Saha B. Chakraborty J. Bhattacharyya S. Chattopadhyay S. Banerjee S. (2008). Contribution of p53-mediated bax transactivation in theaflavin-induced mammary epithelial carcinoma cell apoptosis. Apoptosis 13, 771–781. 10.1007/s10495-008-0213-x 18454316

[B62] Lan C.-Y. Chen S.-Y. Kuo C.-W. Lu C.-C. Yen G.-C. (2019). Quercetin facilitates cell death and chemosensitivity through RAGE/PI3K/AKT/mTOR axis in human pancreatic cancer cells. J. Food Drug Anal. 27, 887–896. 10.1016/j.jfda.2019.07.001 31590760 PMC9306979

[B63] Lee J. Kim J. H. (2016). Kaempferol inhibits pancreatic cancer cell growth and migration through the blockade of EGFR-related pathway *in vitro* . PLoS One 11, e0155264. 10.1371/journal.pone.0155264 27175782 PMC4866780

[B64] Lee S. H. Ryu J. K. Lee K.-Y. Woo S. M. Park J. K. Yoo J. W. (2008). Enhanced anti-tumor effect of combination therapy with gemcitabine and apigenin in pancreatic cancer. Cancer Lett. 259, 39–49. 10.1016/j.canlet.2007.09.015 17967505

[B65] Lee J. Han S.-I. Yun J.-H. Kim J. H. (2015). Quercetin 3-O-glucoside suppresses epidermal growth factor–induced migration by inhibiting EGFR signaling in pancreatic cancer cells. Tumor Biol. 36, 9385–9393. 10.1007/s13277-015-3682-x 26109002

[B66] Lee J. S. Kim Y. R. Song I. G. Ha S.-J. Kim Y. E. Baek N.-I. (2015). Cyanidin-3-glucoside isolated from mulberry fruit protects pancreatic β-cells against oxidative stress-induced apoptosis. Int. J. Mol. Med. 35, 405–412. 10.3892/ijmm.2014.2013 25435295

[B67] Lee J. Kim D.-H. Kim J. H. (2019). Combined administration of naringenin and hesperetin with optimal ratio maximizes the anti-cancer effect in human pancreatic cancer via Down regulation of FAK and p38 signaling pathway. Phytomedicine 58, 152762. 10.1016/j.phymed.2018.11.022 31005717

[B68] Lee K.-S. Lee M.-G. Nam K.-S. (2021). Evaluation of the antimetastatic and anticancer activities of morin in HER2-overexpressing breast cancer SK-BR-3 cells. Oncol. Rep. 46, 126. 10.3892/or.2021.8077 33982761

[B69] Lee W. Song G. Bae H. (2025). Review of synergistic anticancer effects of natural compounds combined with conventional therapeutics against chemoresistance and progression of pancreatic cancer. Mol. Cell. Toxicol. 21, 455–471. 10.1007/s13273-024-00483-1

[B70] Lefort É. C. Blay J. (2013). Apigenin and its impact on gastrointestinal cancers. Mol. Nutr. Food Res. 57, 126–144. 10.1002/mnfr.201200424 23197449

[B71] Lei D. Piao Y. Zhong T. Zhang C. Ai W. Kang Y. (2024). Quercetin attenuates cancer‐associated fibroblast activation via tumor‐stromal interactions and demonstrates its clinical value in pancreatic cancer. J. Food Biochem. 2024, 2177516. 10.1155/2024/2177516

[B72] Li J. Chen X. Kang R. Zeh H. Klionsky D. J. Tang D. (2021). Regulation and function of autophagy in pancreatic cancer. Autophagy 17, 3275–3296. 10.1080/15548627.2020.1847462 33161807 PMC8632104

[B73] Liang C. Wang P. Li M. Li R. Lai K. P. Chen J. (2024). Anti-cancer mechanisms of natural isoflavones against melanoma. Heliyon 10, e28616. 10.1016/j.heliyon.2024.e28616 38586368 PMC10998210

[B74] Liang M.-S. Huang Y. Huang S.-F. Zhao Q. Chen Z.-S. Yang S. (2025). Flavonoids in the treatment of non-small cell lung cancer via immunomodulation: progress to date. Mol. Diagn. Ther. 29, 307–327. 10.1007/s40291-025-00772-y 40036006

[B75] Lin P. Zhang X. Zhu B. Gao J. Yin D. Zeng J. (2023). Naringenin protects pancreatic β cells in diabetic rat through activation of estrogen receptor β. Eur. J. Pharmacol. 960, 176115. 10.1016/j.ejphar.2023.176115 37866740

[B76] Liu Z. Xu W. Han J. Liu Q. Gao L. Wang X. (2020). Quercetin induces apoptosis and enhances gemcitabine therapeutic efficacy against gemcitabine-resistant cancer cells. Anticancer. Drugs 31, 684–692. 10.1097/CAD.0000000000000933 32282368

[B77] Löhr J.-M. Karimi M. Omazic B. Kartalis N. Verbeke C. S. Berkenstam A. (2016). A phase I dose escalation trial of AXP107-11, a novel multi-component crystalline form of genistein, in combination with gemcitabine in chemotherapy-naive patients with unresectable pancreatic cancer. Pancreatology 16, 640–645. 10.1016/j.pan.2016.05.002 27234064

[B78] Lou C. Zhang F. Yang M. Zhao J. Zeng W. Fang X. (2012). Naringenin decreases invasiveness and metastasis by inhibiting TGF-β-Induced epithelial to mesenchymal transition in pancreatic cancer cells. PLoS One 7, e50956. 10.1371/journal.pone.0050956 23300530 PMC3530567

[B79] Ma J. Zeng F. Ma C. Pang H. Fang B. Lian C. (2016). Synergistic reversal effect of epithelial-to-mesenchymal transition by miR-223 inhibitor and genistein in gemcitabine-resistant pancreatic cancer cells. Am. J. Cancer Res. 6, 1384–1395. Available online at: https://pmc.ncbi.nlm.nih.gov/articles/PMC4937740/ (Accessed July 15, 2026). 27429851 PMC4937740

[B80] Mack S. Koessler T. Bichard P. Frossard J.-L. (2025). Pancreatic cancer: epidemiology, risk factors, and prevention. Onco 5, 37. 10.3390/onco5030037

[B81] Malik S. Suchal K. Khan S. I. Bhatia J. Kishore K. Dinda A. K. (2017). Apigenin ameliorates streptozotocin-induced diabetic nephropathy in rats via MAPK-NF-κB-TNF-α and TGF-β1-MAPK-fibronectin pathways. Am. J. Physiol. Physiol. 313, F414–F422. 10.1152/ajprenal.00393.2016 28566504

[B82] McGuigan A. Kelly P. Turkington R. C. Jones C. Coleman H. G. McCain R. S. (2018). Pancreatic cancer: a review of clinical diagnosis, epidemiology, treatment and outcomes. World J. Gastroenterol. 24, 4846–4861. 10.3748/wjg.v24.i43.4846 30487695 PMC6250924

[B83] McMillan B. Riggs D. R. Jackson B. J. Cunningham C. McFadden D. W. (2007). Dietary influence on pancreatic cancer growth by catechin and inositol hexaphosphate. J. Surg. Res. 141, 115–119. 10.1016/j.jss.2007.03.065 17574044

[B84] Mehtsun W. T. Hashimoto D. A. Ferrone C. R. (2019). Status of 5-Year survivors of the whipple procedure for pancreatic adenocarcinoma. Adv. Surg. 53, 253–269. 10.1016/j.yasu.2019.04.012 31327451

[B85] Michel O. Przystupski D. Saczko J. Szewczyk A. Niedzielska N. Rossowska J. (2018). The favourable effect of catechin in electrochemotherapy in human pancreatic cancer cells. Acta Biochim. Pol. 65, 173–184. 10.18388/abp.2018_2602 29796442

[B86] Mir I. A. Tiku A. B. (2015). Chemopreventive and therapeutic potential of “naringenin,” a flavanone present in citrus fruits. Nutr. Cancer 67, 27–42. 10.1080/01635581.2015.976320 25514618

[B87] Mizrahi J. D. Surana R. Valle J. W. Shroff R. T. (2020). Pancreatic cancer. Lancet 395, 2008–2020. 10.1016/S0140-6736(20)30974-0 32593337

[B88] Moeng S. Son S. W. Seo H. A. Lee J. S. Kim C. K. Kuh H. J. (2020). Luteolin-regulated MicroRNA-301-3p targets Caspase-8 and modulates TRAIL sensitivity in PANC-1 cells. Anticancer Res. 40, 723–731. 10.21873/anticanres.14003 32014914

[B89] Montalvo-Javé E. E. Nuño-Lámbarri N. López-Sánchez G. N. Ayala-Moreno E. A. Gutierrez-Reyes G. Beane J. (2023). Pancreatic cancer: genetic conditions and epigenetic alterations. J. Gastrointest. Surg. 27, 1001–1010. 10.1007/s11605-022-05553-0 36749558

[B90] Mostafa H. Behrendt I. Meroño T. González-Domínguez R. Fasshauer M. Rudloff S. (2023). Plasma anthocyanins and their metabolites reduce *in vitro* migration of pancreatic cancer cells, PANC-1, in a FAK- and NF-kB dependent manner: results from the ATTACH-Study a randomized, controlled, crossover trial in healthy subjects. Biomed. Pharmacother. 158, 114076. 10.1016/j.biopha.2022.114076 36516693

[B91] Motallebi M. Bhia M. Rajani H. F. Bhia I. Tabarraei H. Mohammadkhani N. (2022). Naringenin: a potential flavonoid phytochemical for cancer therapy. Life Sci. 305, 120752. 10.1016/j.lfs.2022.120752 35779626

[B92] Mueller S. Engleitner T. Maresch R. Zukowska M. Lange S. Kaltenbacher T. (2018). Evolutionary routes and KRAS dosage define pancreatic cancer phenotypes. Nature 554, 62–68. 10.1038/nature25459 29364867 PMC6097607

[B93] Muhammad N. Usmani D. Tarique M. Naz H. Ashraf M. Raliya R. (2022). The role of natural products and their multitargeted approach to treat solid cancer. Cells 11, 2209. 10.3390/cells11142209 35883653 PMC9318484

[B94] Murthy D. Attri K. S. Singh P. K. (2018). Phosphoinositide 3-Kinase signaling pathway in pancreatic ductal adenocarcinoma progression, pathogenesis, and therapeutics. Front. Physiol. 9, 335. 10.3389/fphys.2018.00335 29670543 PMC5893816

[B95] Naeem A. Hu P. Yang M. Zhang J. Liu Y. Zhu W. (2022). Natural products as anticancer agents: current status and future perspectives. Molecules 27, 8367. 10.3390/molecules27238367 36500466 PMC9737905

[B96] Nimmakayala R. K. Ogunleye A. O. Parte S. Krishna Kumar N. Raut P. Varadharaj V. (2022). PAF1 cooperates with YAP1 in metaplastic ducts to promote pancreatic cancer. Cell Death Dis. 13, 839. 10.1038/s41419-022-05258-x 36180487 PMC9525575

[B97] Nöthlings U. Murphy S. P. Wilkens L. R. Henderson B. E. Kolonel L. N. (2007). Flavonols and pancreatic cancer risk: the multiethnic cohort study. Am. J. Epidemiol. 166, 924–931. 10.1093/aje/kwm172 17690219

[B98] Nwaeburu C. C. Bauer N. Zhao Z. Abukiwan A. Gladkich J. Benner A. (2016). Up-regulation of microRNA let-7c by quercetin inhibits pancreatic cancer progression by activation of numbl. Oncotarget 7, 58367–58380. 10.18632/oncotarget.11122 27521217 PMC5295436

[B99] Oh U. H. Kim D.-H. Lee J. Han S.-I. Kim J.-H. (2022). Eriodictyol induces apoptosis via regulating phosphorylation of JNK, ERK, and FAK/AKT in pancreatic cancer cells. J. Appl. Biol. Chem. 65, 83–88. 10.3839/jabc.2022.011

[B100] Ozcan C. Yaman M. (2015). Determination of myricetin in medicinal plants by high-performance liquid chromatography. Instrum. Sci. Technol. 43, 44–52. 10.1080/10739149.2014.940533

[B101] Pan D. Li S. Zhong P. Chen L. Fang B. Feng Y. (2026). Integrative analysis reveals luteolin’s molecular targets and mechanisms in pancreatic cancer treatment. Eur. J. Med. Res. 31, 570. 10.1186/s40001-026-04056-x 41803987

[B102] Pandey P. Khan F. (2021). A mechanistic review of the anticancer potential of hesperidin, a natural flavonoid from citrus fruits. Nutr. Res. 92, 21–31. 10.1016/j.nutres.2021.05.011 34273640

[B103] Pandey P. Lakhanpal S. Mahmood D. Kang H. N. Kim B. Kang S. (2025). An updated review summarizing the anticancer potential of flavonoids via targeting NF-kB pathway. Front. Pharmacol. 15, 1513422. 10.3389/fphar.2024.1513422 39834817 PMC11743680

[B104] Panzuto F. Boninsegna L. Fazio N. Campana D. Pia Brizzi M. Capurso G. (2011). Metastatic and locally advanced pancreatic endocrine carcinomas: analysis of factors associated with disease progression. J. Clin. Oncol. 29, 2372–2377. 10.1200/JCO.2010.33.0688 21555696

[B105] Paredes-Moscosso S. R. Nathwani A. C. (2024). 10 years of BiTE immunotherapy: an overview with a focus on pancreatic cancer. Front. Oncol. 14, 1429330. 10.3389/fonc.2024.1429330 39759138 PMC11696039

[B106] Park E.-J. Pezzuto M. (2012). Flavonoids in cancer prevention. Anticancer. Agents Med. Chem. 12, 836–851. 10.2174/187152012802650075 22292763

[B108] Park H. J. Choi Y. J. Lee J. H. Nam M. J. (2017). Naringenin causes ASK1-induced apoptosis via reactive oxygen species in human pancreatic cancer cells. Food Chem. Toxicol. 99, 1–8. 10.1016/j.fct.2016.11.008 27838343

[B109] Passero F. C. Saif M. W. (2017). Second line treatment options for pancreatic cancer. Expert Opin. Pharmacother. 18, 1607–1617. 10.1080/14656566.2017.1369955 28820270

[B110] Pavithra R. Khan M. R. Khan M. S. (2024). Recent advancements in natural compounds for cancer therapy and prevention. Phytochem. Rev. 23, 1835–1859. 10.1007/s11101-024-09940-0

[B111] Perumal N. Perumal M. Halagowder D. Sivasithamparam N. (2017a). Morin attenuates diethylnitrosamine-induced rat liver fibrosis and hepatic stellate cell activation by co-ordinated regulation of hippo/yap and TGF-β1/Smad signaling. Biochimie 140, 10–19. 10.1016/j.biochi.2017.05.017 28552397

[B112] Perumal N. Perumal M. Kannan A. Subramani K. Halagowder D. Sivasithamparam N. (2017b). Morin impedes Yap nuclear translocation and fosters apoptosis through suppression of Wnt/β-catenin and NF-κB signaling in Mst1 overexpressed HepG2 cells. Exp. Cell Res. 355, 124–141. 10.1016/j.yexcr.2017.03.062 28366538

[B113] Phillips P. A. Sangwan V. Borja-Cacho D. Dudeja V. Vickers S. M. Saluja A. K. (2011). Myricetin induces pancreatic cancer cell death via the induction of apoptosis and inhibition of the phosphatidylinositol 3-kinase (PI3K) signaling pathway. Cancer Lett. 308, 181–188. 10.1016/j.canlet.2011.05.002 21676539 PMC3126884

[B114] Prabhu L. Mundade R. Korc M. Loehrer P. J. Lu T. (2014). Critical role of NF-κB in pancreatic cancer. Oncotarget 5, 10969–10975. 10.18632/oncotarget.2624 25473891 PMC4294354

[B115] Principe D. R. Underwood P. W. Korc M. Trevino J. G. Munshi H. G. Rana A. (2021). The current treatment paradigm for pancreatic ductal adenocarcinoma and barriers to therapeutic efficacy. Front. Oncol. 11, 688377. 10.3389/fonc.2021.688377 34336673 PMC8319847

[B116] Qanungo S. Das M. Haldar S. Basu A. (2005). Epigallocatechin-3-gallate induces mitochondrial membrane depolarization and caspase-dependent apoptosis in pancreatic cancer cells. Carcinogenesis 26, 958–967. 10.1093/carcin/bgi040 15705601

[B117] Rauf A. Wilairatana P. Joshi P. B. Ahmad Z. Olatunde A. Hafeez N. (2024). Revisiting luteolin: an updated review on its anticancer potential. Heliyon 10, e26701. 10.1016/j.heliyon.2024.e26701 38455556 PMC10918152

[B118] Rawla P. Sunkara T. Gaduputi V. (2019). Epidemiology of pancreatic cancer: global trends, etiology and risk factors. World J. Oncol. 10, 10–27. 10.14740/wjon1166 30834048 PMC6396775

[B119] Rebelo R. Xavier C. P. R. Giovannetti E. Vasconcelos M. H. (2023). Fibroblasts in pancreatic cancer: molecular and clinical perspectives. Trends Mol. Med. 29, 439–453. 10.1016/j.molmed.2023.03.002 37100646

[B121] Rossi M. Lugo A. Lagiou P. Zucchetto A. Polesel J. Serraino D. (2012). Proanthocyanidins and other flavonoids in relation to pancreatic cancer: a case-control study in Italy. Ann. Oncol. Off. J. Eur. Soc. Med. Oncol. 23, 1488–1493. 10.1093/annonc/mdr475 22052986

[B122] Saadh M. J. Mustafa M. A. Malathi H. Ahluwalia G. Kaur S. Al-Dulaimi M. A. A. H. (2024). Targeting the pancreatic tumor microenvironment by plant-derived products and their nanoformulations. Med. Oncol. 41, 201. 10.1007/s12032-024-02443-0 39001987

[B123] Safdar M. A. Aslam R. M. N. Shakeel A. Waqar M. Jmail A. (2023). Cyanidin as potential anticancer agent targeting various proliferative pathways. Chem. Biol. Drug Des. 101, 438–452. 10.1111/cbdd.14173 36326796

[B124] Sahai E. Astsaturov I. Cukierman E. DeNardo D. G. Egeblad M. Evans R. M. (2020). A framework for advancing our understanding of cancer-associated fibroblasts. Nat. Rev. Cancer 20, 174–186. 10.1038/s41568-019-0238-1 31980749 PMC7046529

[B125] Saiki Y. Jiang C. Ohmuraya M. Furukawa T. (2021). Genetic mutations of pancreatic cancer and genetically engineered mouse models. Cancers (Basel) 14, 71. 10.3390/cancers14010071 35008235 PMC8750056

[B126] Saleem H. Anwar S. Alafnan A. Ahemad N. (2021). “Luteolin,” in A Centum of Valuable Plant Bioactives (Elsevier), 509–523. 10.1016/B978-0-12-822923-1.00022-4

[B127] Shankar S. Ganapathy S. Hingorani S. R. Srivastava R. K. (2008). EGCG inhibits growth, invasion, angiogenesis and metastasis of pancreatic cancer. Front. Biosci. 13, 440–452. 10.2741/2691 17981559

[B128] Sharma V. Sharma M. Semwal A. Arora A. Bansal S. Sangwan N. (2026). Morin, a natural flavonoid, activates the PINK1/Parkin pathway to induce mitophagy, promote apoptosis, and suppress cancer stemness in pancreatic cancer. Phyther. Res. 40, 582–602. 10.1002/ptr.70149 41408700

[B129] Sherman M. H. Beatty G. L. (2023). Tumor microenvironment in pancreatic cancer pathogenesis and therapeutic resistance. Annu. Rev. Pathol. Mech. Dis. 18, 123–148. 10.1146/annurev-pathmechdis-031621-024600 PMC9877114 36130070

[B130] Shi S. Wang K. Zhong R. Cassidy A. Rimm E. B. Nimptsch K. (2023). Flavonoid intake and survival after diagnosis of colorectal cancer: a prospective study in 2 US cohorts. Am. J. Clin. Nutr. 117, 1121–1129. 10.1016/j.ajcnut.2023.03.026 37011765 PMC10447476

[B131] Singla N. Gupta G. Kulshrestha R. Sharma K. Bhat A. A. Mishra R. (2024). Daidzein in traditional Chinese medicine: a deep dive into its ethnomedicinal and therapeutic applications. Pharmacol. Res. - Mod. Chin. Med. 12, 100460. 10.1016/j.prmcm.2024.100460

[B132] Sithara T. Arun K. B. Syama H. P. Reshmitha T. R. Nisha P. (2017). Morin inhibits proliferation of SW480 colorectal cancer cells by inducing apoptosis mediated by reactive oxygen species formation and uncoupling of warburg effect. Front. Pharmacol. 8, 640. 10.3389/fphar.2017.00640 28955240 PMC5601037

[B133] Song C. Zheng W. Song C. Zhou H. Yao J. (2024). Protective effects of food-derived kaempferol on pancreatic β-Cells in type 1 diabetes mellitus. Foods 13, 3797. 10.3390/foods13233797 39682869 PMC11640530

[B134] Stefanoudakis D. Frountzas M. Schizas D. Michalopoulos N. V. Drakaki A. Toutouzas K. G. (2024). Significance of TP53, CDKN2A, SMAD4 and KRAS in pancreatic cancer. Curr. Issues Mol. Biol. 46, 2827–2844. 10.3390/cimb46040177 38666907 PMC11049225

[B135] Stern L. Boehme L. Goetz M. Nitschke C. Giannou A. Zhang T. (2022). Potential role of the bitter taste receptor T2R14 in the prolonged survival and enhanced chemoresponsiveness induced by apigenin. Int. J. Oncol. 62, 6. 10.3892/ijo.2022.5454 36382671 PMC9728555

[B136] Suantawee T. Thilavech T. Cheng H. Adisakwattana S. (2020). Cyanidin attenuates methylglyoxal-induced oxidative stress and apoptosis in INS-1 pancreatic β-Cells by increasing Glyoxalase-1 activity. Nutrients 12, 1319. 10.3390/nu12051319 32384625 PMC7284759

[B137] Suhail M. Parveen A. Husain A. Rehan M. (2019). Exploring inhibitory mechanisms of green tea catechins as inhibitors of a cancer therapeutic target, nuclear Factor-κB (NF-κB). Biosci. Biotechnol. Res. Asia 16, 715–723. 10.13005/bbra/2787

[B138] Suhail M. Tarique M. Muhammad N. Naz H. Hafeez A. Zughaibi T. A. (2021). A critical transcription factor NF-κB as a cancer therapeutic target and its inhibitors as cancer treatment options. Curr. Med. Chem. 28, 4117–4132. 10.2174/0929867327666201111142307 33176636

[B139] Suhail M. Rehan M. Tarique M. Tabrez S. Husain A. Zughaibi T. A. (2023). Targeting a transcription factor NF-κB by green tea catechins using *in silico* and *in vitro* studies in pancreatic cancer. Front. Nutr. 9, 1078642. 10.3389/fnut.2022.1078642 36712528 PMC9874859

[B140] Suhail M. Tabrez S. Tarique M. Muhammad N. Rehan M. Zughaibi T. A. (2025). Unlocking the therapeutic potential of polyphenols: promising advances and future directions in pancreatic cancer treatment. Semin. Oncol. 52, 152353. 10.1016/j.seminoncol.2025.152353 40553980

[B141] Treml J. Šmejkal K. (2016). Flavonoids as potent scavengers of hydroxyl radicals. Compr. Rev. Food Sci. Food Saf. 15, 720–738. 10.1111/1541-4337.12204 33401843

[B142] Trivedi A. Hasan A. Ahmad R. Siddiqui S. Srivastava A. Misra A. (2024). Flavonoid myricetin as potent anticancer agent: a possibility towards development of potential anticancer nutraceuticals. Chin. J. Integr. Med. 30, 75–84. 10.1007/s11655-023-3701-5 37340205

[B143] Villalobos-Ayala K. Luongo J. Marsh A. Areas J. Miller B. Williamson T. (2020a). Apigenin modulates immune checkpoint molecules in pancreatic cancer enhancing anti-tumor immunity. J. Immunol. 204, 24117. 10.4049/jimmunol.204.Supp.241.17

[B144] Villalobos-Ayala K. Ortiz Rivera I. Alvarez C. Husain K. DeLoach D. Krystal G. (2020b). Apigenin increases SHIP-1 expression, promotes tumoricidal macrophages and anti-tumor immune responses in murine pancreatic cancer. Cancers (Basel) 12, 3631. 10.3390/cancers12123631 33291556 PMC7761852

[B145] Wan Q. Ren Q. Qiao S. Lyu A. He X. Li F. (2024). Therapeutic potential of flavonoids from traditional Chinese medicine in pancreatic cancer treatment. Front. Nutr. 11, 1477140. 10.3389/fnut.2024.1477140 39650709 PMC11620852

[B146] Wang Z. H. Ah Kang K. Zhang R. Piao M. J. Jo S. H. Kim J. S. (2010). Myricetin suppresses oxidative stress-induced cell damage via both direct and indirect antioxidant action. Environ. Toxicol. Pharmacol. 29, 12–18. 10.1016/j.etap.2009.08.007 21787576

[B147] Wang F. Wang L. Qu C. Chen L. Geng Y. Cheng C. (2021). Kaempferol induces ROS-Dependent apoptosis in pancreatic cancer cells via TGM2-mediated Akt/mTOR signaling. BMC Cancer 21, 396. 10.1186/s12885-021-08158-z 33845796 PMC8042867

[B148] Wen E. Cao Y. He S. Zhang Y. You L. Wang T. (2024). The mitochondria-targeted kaempferol nanoparticle ameliorates severe acute pancreatitis. J. Nanobiotechnology 22, 148. 10.1186/s12951-024-02439-y 38570776 PMC10993609

[B149] Wu D.-G. Yu P. Li J.-W. Jiang P. Sun J. Wang H.-Z. (2014). Apigenin potentiates the growth inhibitory effects by IKK-β-mediated NF-κB activation in pancreatic cancer cells. Toxicol. Lett. 224, 157–164. 10.1016/j.toxlet.2013.10.007 24148603

[B150] Xia J. Cheng L. Mei C. Ma J. Shi Y. Zeng F. (2014). Genistein inhibits cell growth and invasion through regulation of miR-27a in pancreatic cancer cells. Curr. Pharm. Des. 20, 5348–5353. 10.2174/1381612820666140128215756 24479798

[B151] Yamao T. Kai S. Kazami A. Koizumi K. Handa T. Takemoto N. (1999). Tumor markers CEA, CA19-9 and CA125 in monitoring of response to systemic chemotherapy in patients with advanced gastric cancer. Jpn. J. Clin. Oncol. 29, 550–555. 10.1093/jjco/29.11.550 10678558

[B152] Yao D. Cui H. Zhou S. Guo L. (2017). Morin inhibited lung cancer cells viability, growth, and migration by suppressing miR-135b and inducing its target CCNG2. Tumor Biol. 39, 101042831771244. 10.1177/1010428317712443 28975847

[B153] Yildirim M. A. Sevinc B. Paydas S. Karaselek M. A. Duran T. Kuccukturk S. (2025). Exploring the anticancer potential of Dianthus orientalis in pancreatic cancer: a molecular and cellular study. Food Biosci. 66, 106183. 10.1016/j.fbio.2025.106183

[B154] Yu D. Ye T. Xiang Y. Shi Z. Zhang J. Lou B. (2017). Quercetin inhibits epithelial–mesenchymal transition, decreases invasiveness and metastasis, and reverses IL-6 induced epithelial–mesenchymal transition, expression of MMP by inhibiting STAT3 signaling in pancreatic cancer cells. Onco. Targets. Ther. 10, 4719–4729. 10.2147/OTT.S136840 29026320 PMC5626388

[B155] Zhang Y. Liu D. (2011). Flavonol kaempferol improves chronic hyperglycemia-impaired pancreatic beta-cell viability and insulin secretory function. Eur. J. Pharmacol. 670, 325–332. 10.1016/j.ejphar.2011.08.011 21914439

[B156] Zhang Y. Chen A. Y. Li M. Chen C. Yao Q. (2008). Ginkgo biloba extract kaempferol inhibits cell proliferation and induces apoptosis in pancreatic cancer cells. J. Surg. Res. 148, 17–23. 10.1016/j.jss.2008.02.036 18570926 PMC2924764

[B158] Zhang W. Wang J. Gao J. Li H.-L. Han L.-H. Lan Q. (2019). Prediagnostic level of dietary and urinary isoflavonoids in relation to risk of liver cancer in shanghai, China. Cancer Epidemiol. Biomarkers Prev. 28, 1712–1719. 10.1158/1055-9965.EPI-18-1075 31387968 PMC6800062

[B159] Zhang Z. Guo Y. Chen M. Chen F. Liu B. Shen C. (2021). Kaempferol potentiates the sensitivity of pancreatic cancer cells to erlotinib via inhibition of the PI3K/AKT signaling pathway and epidermal growth factor receptor. Inflammopharmacology 29, 1587–1601. 10.1007/s10787-021-00848-1 34322786

[B160] Zhang Z. Wang J. Miao W. Lin Q. Ji H. Li X. (2025). Recent advances in bio-based co-delivery systems for food bioactive compounds: a review. Food Biosci. 63, 105758. 10.1016/j.fbio.2024.105758

[B161] Zhou J. Enewold L. Stojadinovic A. Clifton G. T. Potter J. F. Peoples G. E. (2010). Incidence rates of exocrine and endocrine pancreatic cancers in the United States. Cancer Causes Control 21, 853–861. 10.1007/s10552-010-9512-y 20182788

